# Supplementary material for: Relating local connectivity and global dynamics in recurrent excitatory-inhibitory networks
Source: PLoS Comput Biol. 2023 Jan 23;19(1):e1010855. doi: 10.1371/journal.pcbi.1010855 (PMC9894562; doi:10.1371/journal.pcbi.1010855)
Supplement: S1 Text — (PDF) [file pcbi.1010855.s001.pdf]

Relating local connectivity and global dynamics in recurrent excitatory-inhibitory networks

Yuxiu Shao*, Srdjan Ostojic*

Laboratoire de Neurosciences Cognitives et Computationnelles, INSERM U960, Ecole Normale Superieure - PSL Research University, Paris, France

* yuxiu.shao@ens.psl.eu (YS), * srdjan.ostojic@ens.fr (SO)

Supporting information

S1 Text. Dynamics in Gaussian-mixture low-rank networks. Here, we provide the derivation for the dynamics of the latent variable κ (Eq. (113)) in the Gaussian-mixture low-rank network model. We consider a rank-one connectivity consisting of P populations, with the neurons in each population accounting for a α_p percentage of all neurons. The entries on the left and right eigenvectors $\mathbf{n}^p$, $\mathbf{m}^p$ assigned to population p are sampled from a multivariate Gaussian distribution with mean $\bar{n}^p$, $\bar{m}^p$, variance $\sigma^2_{n^p}$, $\sigma^2_{m^p}$ and covariance σ^p_{nm} (Methods Sec. 2.3) So, κ_{rec} in Eq. (109) is further decomposed into two integrals involving the contributions from the mean and random connected components, respectively,

$$\begin{aligned}\kappa_{rec} = &\sum_{p=1}^{P}\alpha_p \int \mathrm{d}m P^p(m)\bar{n}^p\phi(\kappa m)\\ &+\sum_{p=1}^{P}\alpha_p \int \mathrm{d}m\mathrm{d}n P^p(m,n)(n-\bar{n}^p)\phi(\kappa m),\end{aligned} \tag{132}$$

here, $P^p(m)$ represents the marginal Gaussian distribution for connectivity loadings m_i of neurons in population p

$$P^p(m) = \mathcal{N}(\bar{m}^p, \sigma^2_{m^p}), \tag{133}$$

and $P^p(m,n)$ represents the multivariate Gaussian distribution for connectivity loadings m_i, n_i of neurons in population p (Eq. (108)).

Note that the synaptic input to neurons in population p is a scaled Gaussian variable $x_i = \kappa m_i$ corresponding to the Gaussian loadings $m_i \sim \mathcal{N}(\bar{m}^p, \sigma^2_{m^p})$, so it conforms to a Gaussian distribution $\mathcal{N}(\mu^p_x, \Delta^p_x)$ with the population-averaged mean and variance

$$\begin{aligned}\mu^p_x &= \kappa\bar{m}^p,\\ \Delta^p_x &= \kappa^2\sigma^2_{m^p}.\end{aligned} \tag{134}$$

Thus, the first term in Eq. (132) is

$$\int \mathrm{d}m\mathcal{N}(\bar{m}^p, \sigma^2_{m^p})\phi(\kappa m) = \langle\phi(\kappa\bar{m}^p, \kappa^2\sigma^2_{m^p})\rangle = \langle\phi(\mu^p_x, \Delta^p_x)\rangle, \tag{135}$$

a Gaussian integral term.

We then use Stein's lemma

$$\int \mathcal{D}z z f(z) = \int \mathcal{D}z \frac{\mathrm{d}}{\mathrm{d}z} f(z) \tag{136}$$

and further replace the multivariate Gaussian distribution by Eq. (108), to compute the second term in Eq. (132) attributed to the random connectivity component as

$$\begin{aligned}&\int \mathrm{d}m\mathrm{d}n\mathcal{N}\left(\begin{pmatrix}\bar{m}^p\\ \bar{n}^p\end{pmatrix}, \begin{pmatrix}\sigma^2_{m^p} & \sigma^p_{nm}\\ \sigma^p_{nm} & \sigma^2_{n^p}\end{pmatrix}\right)(n-\bar{n}^p)\phi(\kappa m)\\ &\qquad = \langle\phi(\kappa\bar{m}^p, \kappa^2\sigma^2_{m^p})\rangle\sigma^p_{nm}\kappa\\ &\qquad = \langle\phi(\mu^p_x, \Delta^p_x)\rangle\sigma^p_{nm}\kappa.\end{aligned} \tag{137}$$

Finally, combining the contributions from both the mean and random components, we re-express κ_{rec} in Eq. (109) and retrieve Eq. (113).
