# Supplementary material for: Relating local connectivity and global dynamics in recurrent excitatory-inhibitory networks
Source: PLoS Comput Biol. 2023 Jan 23;19(1):e1010855. doi: 10.1371/journal.pcbi.1010855 (PMC9894562; doi:10.1371/journal.pcbi.1010855)
Supplement: S2 Text — (PDF) [file pcbi.1010855.s002.pdf]

Relating local connectivity and global dynamics in recurrent excitatory-inhibitory networks

Yuxiu Shao*, Srdjan Ostojic*

Laboratoire de Neurosciences Cognitives et Computationnelles, INSERM U960, Ecole Normale Superieure - PSL Research University, Paris, France

* yuxiu.shao@ens.psl.eu (YS), * srdjan.ostojic@ens.fr (SO)

# Supporting information

**S2 Text. Linear stability at fixed points in rank-one networks.** To determine the stability of fixed points of the network with rank-one connectivity structure, we consider the rank-one connectivity $\mathbf{J}_{R1}$ and study the stability of the one-dimensional latent dynamical variable $\kappa$ in the neighbourhood area of its fixed point $\kappa^0$. We set the fixed point of synaptic input $x_i^0$, define the perturbation of the latent variable $\kappa^1$ and the perturbation of the synaptic input $x_i^1 = \kappa^1 m_i$, the temporal evolution of $\kappa^1$ is expressed as

$$\begin{aligned}\dot{\kappa}^1 &= -\kappa^1 + \langle n_i[\phi'(x_i^0)x_i^1]\rangle \\ &= -\kappa^1 + \sum_{p=E,I}\alpha_p\langle n_i^p\phi'(x_i^{p,0})x_i^{p,1}\rangle,\end{aligned} \tag{138}$$

for the rank-one approximation network $\mathbf{J}_{R1} = \mathbf{m}\mathbf{n}^\intercal/N$, we remove symbol $[\cdot]$. Next, in the Gaussian-mixture low-rank framework, the entries $n_i^p$ and $m_i^p$ are jointly sampled from a bivariate Gaussian distribution characterized by means, variances and covariances, $\bar{m}^p$, $\bar{n}^p$ and $\sigma^2_{m^p}$, $\sigma^2_{n^p}$, $\sigma^p_{nm}$. Using a similar approach to previous studies [1,2], we set

$$\begin{aligned}n_i^p &= \bar{n}^p + \big(\sqrt{1-\gamma}\zeta_n^p + \sqrt{\gamma}\zeta^p\big)\sigma_{n^p} \\ m_i^p &= \bar{m}^p + \big(\sqrt{1-\gamma}\zeta_m^p + \sqrt{\gamma}\zeta^p\big)\sigma_{m^p} \\ \sigma^p_{nm} &= \gamma\sigma_{n^p}\sigma_{m^p}\end{aligned} \tag{139}$$

where $\zeta_m^p$, $\zeta_n^p$ and $\zeta^p$ are three independent normal random variables $\mathcal{N}(0,1)$. By substituting variables in Eq. (139), after some linear algebra, we get

$$\begin{aligned}\langle n_i^p\phi'(x_i^{p,0})x_i^{p,1}\rangle &= \langle n_i^p\phi'(x_i^{p,0})\kappa^1 m_i^{p,1}\rangle \\ &= \big((\bar{n}^p\bar{m}^p + \sigma^p_{nm})\langle\phi'\rangle + \bar{n}^p\sigma^2_{m^p}\langle\phi''\rangle\kappa^0 + \bar{m}^p\sigma^p_{nm}\langle\phi''\rangle\kappa^0 \\ &+ \sigma^2_{m^p}\sigma^p_{nm}\langle\phi'''\rangle(\kappa^0)^2\big)\kappa^1.\end{aligned} \tag{140}$$

We finally obtain the time evolution of $\kappa$

$$\begin{aligned}\dot{\kappa}^1 = -\kappa^1 + \sum_{p=E,I}\alpha_p\big((\bar{n}^p\bar{m}^p + \sigma^p_{nm})\langle\phi'\rangle + \bar{n}^p\sigma^2_{m^p}\langle\phi''\rangle\kappa^0 \\ + \bar{m}^p\sigma^p_{nm}\langle\phi''\rangle\kappa^0 + \sigma^2_{m^p}\sigma^p_{nm}\langle\phi'''\rangle(\kappa^0)^2\big)\kappa^1.\end{aligned} \tag{141}$$

The Jacobian for the latent dynamical variable's fixed point $\kappa^0$ is

$$\begin{aligned}S_\kappa = -1 + \sum_{p=E,I}\alpha_p\big((\bar{n}^p\bar{m}^p + \sigma^p_{nm})\langle\phi'\rangle + \bar{n}^p\sigma^2_{m^p}\langle\phi''\rangle\kappa^0 \\ + \bar{m}^p\sigma^p_{nm}\langle\phi''\rangle\kappa^0 + \sigma^2_{m^p}\sigma^p_{nm}\langle\phi'''\rangle(\kappa^0)^2\big),\end{aligned} \tag{142}$$

which is a scalar in our rank-one network. The fixed point is stable when $S_\kappa < 0$ and unstable when $S_\kappa > 0$. All the above parameters are expressed and calculated in the text, so that we can check the stability of each fixation point using the formulas above.

# References

1. Mastrogiuseppe F, Ostojic S. Linking connectivity, dynamics, and computations in low-rank recurrent neural networks. Neuron. 2018;99(3):609–623.

2. Beiran M, Dubreuil A, Valente A, Mastrogiuseppe F, Ostojic S. Shaping dynamics with multiple populations in low-rank recurrent networks. Neural Computation. 2021;33(6):1572–1615.
