# Supplementary material for: Relating local connectivity and global dynamics in recurrent excitatory-inhibitory networks
Source: PLoS Comput Biol. 2023 Jan 23;19(1):e1010855. doi: 10.1371/journal.pcbi.1010855 (PMC9894562; doi:10.1371/journal.pcbi.1010855)
Supplement: S3 Text — (PDF) [file pcbi.1010855.s003.pdf]

Relating local connectivity and global dynamics in recurrent excitatory-inhibitory networks

Yuxiu Shao*, Srdjan Ostojic*

Laboratoire de Neurosciences Cognitives et Computationnelles, INSERM U960, Ecole Normale Superieure - PSL Research University, Paris, France

* yuxiu.shao@ens.psl.eu (YS), * srdjan.ostojic@ens.fr (SO)

Supporting information

S3 Text. Comparison between dynamics in full-rank connectivity with rank-one approximation. Here, we compare the dynamics solved in the full-rank network with dynamics solved by the rank-one approximation, considering the limitations of applying the classical MFT, we only discuss networks with independent connections.

Using the rank-one approximation, global dynamics is characterized by the latent dynamical variable κ, which satisfies

$$\kappa = J_E \langle \phi(\kappa, \kappa^2 \sigma^2_{m^E}) \rangle - J_I \langle \phi(\kappa, \kappa^2 \sigma^2_{m^I}) \rangle. \tag{143}$$

Combining the connectivity statistics given in Eqs. (54)-(56), (90), we thus express the self-consistent equations for the population mean and variance of the synaptic inputs x_i using κ (Eq. (111))

$$\mu_x^E = \mu_x^I = J_E \langle \phi(\mu_x^E, \Delta_x^E) \rangle - J_I \langle \phi(\mu_x^I, \Delta_x^I) \rangle \tag{144}$$

and

$$\begin{aligned} \Delta_x^E &= \frac{1}{\lambda_0^2} \big(J_E \langle \phi(\mu_x^E, \Delta_x^E) \rangle - J_I \langle \phi(\mu_x^I, \Delta_x^I) \rangle \big)^2 \big(\alpha_E g^2_{EE} + \alpha_I g^2_{EI} \big) \\ \Delta_x^I &= \frac{1}{\lambda_0^2} \big(J_E \langle \phi(\mu_x^E, \Delta_x^E) \rangle - J_I \langle \phi(\mu_x^I, \Delta_x^I) \rangle \big)^2 \big(\alpha_E g^2_{IE} + \alpha_I g^2_{II} \big). \end{aligned} \tag{145}$$

Comparing with the self-consistent equations for population mean and variance in the full-rank network Eq. (130), we find in particular that the expression for μ_x^p is the same in both representations, but the expressions for the variance Δ_x^p are different. Specifically, the low-rank approximation shows that the different heterogeneity between excitatory and inhibitory populations depends only on the block-structured variances, i. e. , $\sum_{q=E,I} \alpha_p g^2_{pq}/\lambda_0^2$, and independent of the historical population activity. The full-rank dynamics, on the other hand, shows that the different heterogeneity depends on the local relationship between block structured variance and the structure of historical population activity [1].

For a simplified network example, where the locally defined connectivity has homogeneous random parameters $g_{pq} = g,\ p = E, I$, the variance of the rank-one perturbation eigenvector $\sigma^2_{m^p}$ is thereby the same for both excitatory and inhibitory populations. Because $\lambda_0 = J_E - J_I$, means and variances in Eqs. (144), (145) are

$$\begin{aligned} \mu_x &= \mu_x^E = \mu_x^I = \lambda_0 \langle \phi(\mu_x, \Delta_x) \rangle, \\ \Delta_x &= \Delta_x^E = \Delta_x^I = g^2 \langle \phi(\mu_x, \Delta_x) \rangle^2. \end{aligned} \tag{146}$$

So that, for this simple example, the heterogeneity of dynamics in the full-rank approximation is $g^2 \langle (\phi(\mu_x, \Delta_x))^2 \rangle$ (Eq. (130)), while the heterogeneity in the rank-one approximation is $g^2 \langle \phi(\mu_x, \Delta_x) \rangle^2$, the difference does not substantially change the bistable transition and the performance of the low-rank approximation.

References

1. Valente A, Ostojic S, Pillow J. Probing the relationship between linear dynamical systems and low-rank recurrent neural network models. Neural Computation. 2022;34(9):1871–1892.
