# Supplementary material for: Relating local connectivity and global dynamics in recurrent excitatory-inhibitory networks
Source: PLoS Comput Biol. 2023 Jan 23;19(1):e1010855. doi: 10.1371/journal.pcbi.1010855 (PMC9894562; doi:10.1371/journal.pcbi.1010855)
Supplement: S4 Text — (PDF) [file pcbi.1010855.s004.pdf]

Relating local connectivity and global dynamics in recurrent excitatory-inhibitory networks

Yuxiu Shao*, Srdjan Ostojic*

Laboratoire de Neurosciences Cognitives et Computationnelles, INSERM U960, Ecole Normale Superieure - PSL Research University, Paris, France

* yuxiu.shao@ens.psl.eu (YS), * srdjan.ostojic@ens.fr (SO)

Supporting information

S4 Text. Chaotic dynamical transition point for networks with independent connectivity. Conventionally, the collective dynamics of random neural network becomes chaotic when the effect of random component is too strong. Based on previous study [1], when the effective random gain, also known as the radius of the random eigenvalue bulk r_g, exceed the critical threshold 1, the network enters the chaotic dynamical state.

Considering the block-like E-I random network with i. i. d. random connectivity, we define a $\mathbb{R}^{2\times 2}$ matrix with elements $\alpha_q g_{pq}^2$

$$\mathbf{M}_g = \begin{bmatrix} \alpha_E g_{EE}^2 & \alpha_I g_{EI}^2 \\ \alpha_E g_{IE}^2 & \alpha_I g_{II}^2 \end{bmatrix}, \tag{147}$$

the first eigenvalue of $\mathbf{M}_g$ determines the radius of the continuous eigenvalues bulk of $\mathbf{J}$, that is

$$r_g = \sqrt{\lambda_{M_g}}. \tag{148}$$

For the sparse E-I network, considering the relationships given by Eq. (38), we calculate the radius for the sparse network as

$$r_g = \sqrt{(N_E A_E^2 + N_I A_I^2)c(1-c)}. \tag{149}$$

References

1. Aljadeff J, Stern M, Sharpee T. Transition to chaos in random networks with cell-type-specific connectivity. Physical review letters. 2015;114(8):088101.
