# Supplementary material for: Relating local connectivity and global dynamics in recurrent excitatory-inhibitory networks
Source: PLoS Comput Biol. 2023 Jan 23;19(1):e1010855. doi: 10.1371/journal.pcbi.1010855 (PMC9894562; doi:10.1371/journal.pcbi.1010855)
Supplement: S5 Text — (PDF) [file pcbi.1010855.s005.pdf]

Relating local connectivity and global dynamics in recurrent excitatory-inhibitory networks

Yuxiu Shao*, Srdjan Ostojic*

Laboratoire de Neurosciences Cognitives et Computationnelles, INSERM U960, Ecole Normale Superieure - PSL Research University, Paris, France

* yuxiu.shao@ens.psl.eu (YS), * srdjan.ostojic@ens.fr (SO)

Supporting information

S5 Text. Local connectivity statistics in rank-R Gaussian-mixture models. When a rank-R connectivity matrix satisfies the assumption that for each population where i belongs to, $m_i,\ n_i$ are drawn independently between neurons from a multi-variate Gaussian distribution, we show that in the limit of large network, the statistical properties of the entries on the rank-R connectivity vectors $\bar{m}^{(r),p},\ \bar{n}^{(r),p},\ \sigma^{,}_{m_r^p}\ \sigma^2_{n_r^p}$ and $\sigma^p_{n_r m_{r'}}$ directly determine the means $\bar{J}_{pq}$, variances $\sigma^2_{z_{pq}}$ and reciprocal correlations η_{pq} of the resulting local synaptic weights J_{ij}, where $i \in N_p,\ j \in N_q$. Considering Eq. (2), the cell-type-dependent mean

$$\bar{J}_{pq} = \frac{1}{N}\sum_{r=1}^{R} \bar{m}_i^{(r)}\bar{n}_j^{(r)} = \frac{1}{N}\sum_{r=1}^{R}\bar{m}^{(r),p}\bar{n}^{(r),q}, \tag{150}$$

and the cell-type-dependent variance of locally defined connections is

$$\begin{aligned}
\sigma^2_{z_{pq}} &= \frac{1}{N^2}\Big[\big(\sum_{r=1}^{R} m_i^{(r)} n_j^{(r)} - \sum_{r=1}^{R}\bar{m}^{(r),p}\bar{n}^{(r),q}\big)^2\Big] \\
&= \frac{1}{N^2}\Big[\big(\sum_{r=1}^{R}\Delta m_i^{(r)}\bar{n}^{(r),q} + \bar{m}^{(r),p}\Delta n_j^{(r)} + \Delta m_i^{(r)}\Delta n_j^{(r)}\big)^2\Big] \\
&= \frac{1}{N^2}\sum_{r=1}^{R}\big(\sigma^2_{m_r^p}(\bar{n}^{(r),q})^2 + (\bar{m}^{(r),p})^2\sigma^2_{n_r^q} + \sigma^2_{m_r^p}\sigma^2_{n_r^q}\big).
\end{aligned} \tag{151}$$

Finally the correlation between the pairwise weights $J_{ij},\ J_{ji}$ is computed as

$$\begin{aligned}
&\big[(J_{ij} - [J_{ij}])(J_{ji} - [J_{ji}])\big]_J \\
&= \frac{1}{N^2}\Big[\sum_{r,r'=1}^{R} m_i^{(r)} n_j^{(r)} m_j^{(r')} n_i^{(r')} - \sum_{r,r'=1}^{R} m_i^{(r)} n_j^{(r)}\bar{m}^{(r'),q}\bar{n}^{(r'),p} \\
&- \sum_{r,r'=1}^{R}\bar{m}^{(r),p}\bar{n}^{(r),q} m_j^{(r')} n_i^{(r')} + \sum_{r,r'=1}^{R}\bar{m}^{(r),p}\bar{n}^{(r),q}\bar{m}^{(r'),q}\bar{n}^{(r'),p}\Big] \\
&= \frac{1}{N^2}\sum_{r,r'=1}^{R}\big(\sigma^p_{n_{r'}m_r}\bar{m}^{(r'),q}\bar{n}^{(r),q} + \sigma^q_{n_r m_{r'}}\bar{m}^{(r),p}\bar{n}^{(r'),p} + \sigma^q_{n_r m_{r'}}\sigma^p_{n_{r'}m_r}\big).
\end{aligned} \tag{152}$$

Substituting Eqs. (151), (152) into Eq. (31) leads to the resulting reciprocal correlation η_{pq}.
