# Supplementary material for: Relating local connectivity and global dynamics in recurrent excitatory-inhibitory networks
Source: PLoS Comput Biol. 2023 Jan 23;19(1):e1010855. doi: 10.1371/journal.pcbi.1010855 (PMC9894562; doi:10.1371/journal.pcbi.1010855)
Supplement: S6 Text — (PDF) [file pcbi.1010855.s006.pdf]

Relating local connectivity and global dynamics in recurrent excitatory-inhibitory networks

Yuxiu Shao*, Srdjan Ostojic*

Laboratoire de Neurosciences Cognitives et Computationnelles, INSERM U960, Ecole Normale Superieure - PSL Research University, Paris, France

* yuxiu.shao@ens.psl.eu (YS), * srdjan.ostojic@ens.fr (SO)

Supporting information

S6 Text. First-order approximation of eigenvalues and eigenvectors. Here we provide nonrigorous but intuitive derivations of our approximation of eigenvalues and eigenvectors, together with a light discussion of the approximation's limitations and restrictions.

Eigenvalue outliers. The solutions of the determinant lemma Eqs. (59), (60) are the eigenvalues of $\bar{\mathbf{m}}\bar{\mathbf{n}}^\intercal/N + \mathbf{Z}$, as long as $(\mathbf{I} - \mathbf{Z}/\lambda)$ is invertible and λ is not the eigenvalue of $\mathbf{Z}$. Because we are focusing on the outlying eigenvalue that is larger than the spectral radius on the real axis r_g, this can be presumably translated into that the norm of λ of the low-rank part $\mathbf{m}\mathbf{n}^\intercal/N$ is larger than the spectral norm of the random part $\mathbf{Z}$, which gives the upper bound on its spectral radius [1]. We can then expand $(\mathbf{I} - \mathbf{Z}/\lambda)^{-1}$ in series, and get Eq. (7) (Eq. (61)) from Eq. (60).

Despite the fact that Eq. (7) is a polynomial with infinite terms, there are at most finite N solutions for the eigenvalue outliers in general, where N is the size of the network [2]. In particular, in this work, we assume that second-order reciprocal motifs are the only non-trivial random structures in the network connectivity. Therefore, only the terms with even $k = 0,\ 2, \ldots$ are non-zero in the large network limit after averaging over realizations of the random matrix $\mathbf{Z}$ (Eq. (7), Methods Sec. 2.3). And we get

$$[\lambda] = \frac{1}{N}\bar{\mathbf{n}}^\intercal \sum_{l=0}^{\infty}\left[\frac{\mathbf{Z}^{2l}}{\lambda^{2l}}\right]\bar{\mathbf{m}}. \tag{153}$$

In general, as $[\mathbf{Z}^k] \neq [\mathbf{Z}]^k$ for $k > 1$, there is no trivial resummation in Eq. (153). However, we assume that there are only reciprocal motifs and no other random structures, so we approximate the even power term $[\mathbf{Z}^{2l}/\lambda^{2l}] \approx [\mathbf{Z}^2/\lambda^2]^l$, and the numerical simulations show that this approximation is accurate for the scenarios we examined. We therefore get the resummation of the geometric series as

$$\sum_{l=0}^{\infty}\left[\frac{\mathbf{Z}^{2l}}{\lambda^{2l}}\right] \approx \sum_{l=0}^{\infty}\left[\frac{\mathbf{Z}^{2}}{\lambda^{2}}\right]^l = \left(\mathbf{I} - \left[\frac{\mathbf{Z}^{2}}{\lambda^{2}}\right]\right)^{-1} \tag{154}$$

under the constraint that $\|\mathbf{Z}\|/\|\lambda\| < 1$. For the networks with i. i. d. Gaussian random synaptic weights, $[\mathbf{Z}^2] = 0$ in the large network limit, thereby we easily get the result $[\lambda] = \lambda_0$. For the networks with reciprocal Gaussian random connections, $[\mathbf{Z}^2]$ becomes a diagonal matrix, the diagonal elements of the matrix are computed as

$$\begin{aligned}
\psi^E &= \Big[\sum_{j=1}^{N} z_{ij}z_{ji}\Big] = \alpha_E g_{EE}^2\eta_{EE} + \alpha_I g_{EI}g_{IE}\eta_{EI} + \mathcal{O}(1/N), \quad i \in N_E \\
\psi^I &= \Big[\sum_{j=1}^{N} z_{ij}z_{ji}\Big] = \alpha_E g_{EI}g_{IE}\eta_{EI} + \alpha_I g_{II}^2\eta_{II} + \mathcal{O}(1/N), \qquad i \in N_I,
\end{aligned} \tag{155}$$

in the limit $N \to \infty$, the right-most terms for $\psi^E,\ \psi^I$ become zero. The inverse matrix is

$$\left(\mathbf{I} - \left[\frac{\mathbf{Z}^2}{\lambda^2}\right]\right)^{-1} = \begin{bmatrix} \frac{[\lambda^2]}{[\lambda^2]-\psi^E} & & & \\ & \ddots & & \\ & & \frac{[\lambda^2]}{[\lambda^2]-\psi^I} & \\ & & & \ddots \end{bmatrix} \tag{156}$$

We substitute the approximation Eq. (156) into Eq. (153) and get the fourth-order polynomial equation for $[\lambda]$ as

$$\begin{aligned} f([\lambda]) = [\lambda]^4 - (J_E - J_I)[\lambda]^3 - (\psi^E + \psi^I)[\lambda]^2 \\ + (J_E\psi^I - J_I\psi^E)[\lambda] + \psi^E\psi^I = 0. \end{aligned} \tag{157}$$

We take a network connectivity with homogeneous variance g^2/N and reciprocal correlation η as an example. Then $\psi_E = \psi_I = g^2\eta$ at the large network limit, and Eq. (157) is simply re-written as

$$\begin{aligned} f([\lambda]) &= [\lambda]^4 - \lambda_0[\lambda]^3 - 2g^2\eta[\lambda]^2 + \lambda_0 g^2\eta[\lambda] + g^4\eta^2 \\ &= ([\lambda]^2 - g^2\eta)([\lambda]^2 - \lambda_0[\lambda] - g^2\eta) = 0 \end{aligned} \tag{158}$$

where $\lambda_0 = J_E - J_I$. Solving the above equation we get

$$\lambda_{1,2} = \frac{\lambda_0 \pm \sqrt{\lambda_0^2 + 4g^2\eta}}{2}, \quad s.\ t. \quad \eta > -\frac{\lambda_0^2}{4g^2} \tag{159}$$

and

$$\lambda_{3,4} = \pm g\sqrt{\eta}, \quad s.\ t. \quad \eta > 0. \tag{160}$$

In the main text, we truncate the series formula Eq. (7) for eigenvalue λ, and keep k up to 2, since the term with θ_2 is the first term to manifest the non-trivial impacts of the reciprocal motifs onto the eigenvalues. Considering the corresponding infinite series sum formula Eq. (154), we then compute the ratio between the resumming of $l \geq 2$ ($k > 3$ in Eq. (7)) $\sum_{l=2}^{\infty}[\mathbf{Z}^2/\lambda^2]^l$ and the summing of infinite series $\sum_{l=0}^{\infty}[\mathbf{Z}^2/\lambda^2]^l$

$$ratio = \frac{\sum_{l=2}^{\infty}[\mathbf{Z}^2/\lambda^2]^l}{\sum_{l=0}^{\infty}[\mathbf{Z}^2/\lambda^2]^l} = \left[\frac{\mathbf{Z}^2}{\lambda^2}\right]^2. \tag{161}$$

Non-zero diagonal elements on $[\mathbf{Z^2}]$ are on the same order as g_{pq}^2 and are comparable to the square of the spectral radius of the eigenvalue bulk (Eqs. (147), (148)), as we have shown in Eqs. (86), (155). Since we are assuming that this radius is smaller than the norm of the eigenvalue λ, $ratio = [\mathbf{Z}^2/\lambda^2]^2$ is sufficiently small. The terms where $k > 2$ have little impact on predicting the outlier corresponding to the dominant low-rank real component. For all example networks with reciprocal motifs, we compare the outlier predictions obtained from truncation approximation and infinite series summing (Figs 3, 4 and 9), and we demonstrate that the truncation approximation predicts λ as accurately as the infinite series summing.

In the truncation approximation for eigenvalue outlier, the coefficient $\theta_2 = \bar{\mathbf{n}}^\intercal\mathbf{Z}^2\bar{\mathbf{m}}/N$ in particular appears to link to the overlap of low-rank vectors $\sigma_{nm} = \bar{\mathbf{n}}^\intercal\mathbf{Z}^2\bar{\mathbf{m}}/(N\lambda_0^2)$, which further provides us with a better understanding of how reciprocal motifs influence the dynamics by controlling the dominant outlier.

Eigenvectors. Next, we elaborate the derivations of the perturbations of eigenvectors [3]. We define $\bar{\mathbf{L}},\ \bar{\mathbf{R}}$ and λ_0 as the non-trivial left, right eigenvectors and eigenvalue of the mean matrix $\bar{\mathbf{J}}$ (Eq. (52)). We further define $\bar{\mathbf{L}}_1,\ \bar{\mathbf{R}}_1 \in \mathbb{R}^{N\times(N-1)}$, their columns are respectively the bases for the left and right $(N-1)$-dimensional space of $\bar{\mathbf{J}}$, and $\mathbf{B}_1 \in \mathbb{R}^{(N-1)\times(N-1)}$ is the corresponding diagonal eigenvalue matrix. These variables overall satisfy

$$\bar{\mathbf{L}}^\intercal\bar{\mathbf{R}} = 1, \quad \bar{\mathbf{L}}_1^\intercal\bar{\mathbf{R}}_1 = \mathbf{I}_{(N-1)}, \quad [\bar{\mathbf{L}},\ \bar{\mathbf{L}}_1]^\intercal\bar{\mathbf{J}}[\bar{\mathbf{R}},\ \bar{\mathbf{R}}_1] = \begin{bmatrix} \lambda_0 & \mathbf{0} \\ \mathbf{0} & \mathbf{B}_1 \end{bmatrix} \tag{162}$$

in this particular case $\bar{\mathbf{J}}$, $\mathbf{B}_1 = \mathbf{0}$. We furthermore define the dominant left and right eigenvectors of the perturbing matrix $\mathbf{J} = \bar{\mathbf{J}} + \mathbf{Z}$ as $\mathbf{L},\ \mathbf{R}$, as well as the corresponding eigenvalue as λ. We differentiate the equation $\mathbf{J}\mathbf{R} = \lambda\mathbf{R}$, that is $\mathbf{Z}\bar{\mathbf{R}} + \bar{\mathbf{J}}\mathbf{R}' = \lambda'\bar{\mathbf{R}} + \lambda_0\mathbf{R}'$ and obtain

$$(\mathbf{Z} - \lambda'\mathbf{I}_N)\bar{\mathbf{R}} = -(\bar{\mathbf{J}} - \lambda_0\mathbf{I}_N)\mathbf{R}'. \tag{163}$$

We use the Lemma

$$\bar{\mathbf{J}} - \lambda_0\mathbf{I}_N = [\bar{\mathbf{R}},\ \bar{\mathbf{R}}_1]\begin{bmatrix}\mathbf{0} & \mathbf{0}\\ \mathbf{0} & \mathbf{B}_1 - \lambda_0\mathbf{I}_{N-1}\end{bmatrix}[\bar{\mathbf{L}},\ \bar{\mathbf{L}}_1]^\intercal, \tag{164}$$

substituting this equation into Eq. (163) and multiplying $[\bar{\mathbf{L}},\ \bar{\mathbf{L}}_1]^\intercal$ on the left, and get

$$[\bar{\mathbf{L}},\ \bar{\mathbf{L}}_1]^\intercal(\mathbf{Z} - \lambda'\mathbf{I}_N)\bar{\mathbf{R}} = -\begin{bmatrix}\mathbf{0} & \mathbf{0}\\ \mathbf{0} & \mathbf{B}_1 - \lambda_0\mathbf{I}_{N-1}\end{bmatrix}[\bar{\mathbf{L}},\ \bar{\mathbf{L}}_1]^\intercal\mathbf{R}'. \tag{165}$$

We remove the first row equation using the formula for λ'

$$\lambda' = \bar{\mathbf{L}}^\intercal\mathbf{Z}\bar{\mathbf{R}}, \tag{166}$$

and simplify the remaining equations as

$$\bar{\mathbf{L}}_1^\intercal(\mathbf{Z} - \lambda'\mathbf{I}_N)\bar{\mathbf{R}} = -(\mathbf{B}_1 - \lambda_0\mathbf{I}_{N-1})\bar{\mathbf{L}}_1^\intercal\mathbf{R}', \tag{167}$$

further

$$\bar{\mathbf{L}}_1^\intercal\mathbf{R}' = -(\mathbf{B}_1 - \lambda_0\mathbf{I}_{N-1})^{-1}\bar{\mathbf{L}}_1^\intercal\mathbf{Z}\bar{\mathbf{R}}. \tag{168}$$

We then multiply on the left by $\bar{\mathbf{R}}_1$, and using the relationships $\bar{\mathbf{R}}_1\bar{\mathbf{L}}_1^\intercal = \mathbf{I}_N - \bar{\mathbf{R}}\bar{\mathbf{L}}^\intercal$ and $\bar{\mathbf{L}}^\intercal\mathbf{R}' = 0$, after some linear algebra, we get

$$\mathbf{R}' = -\bar{\mathbf{R}}_1(\mathbf{B}_1 - \lambda_0\mathbf{I}_{N-1})^{-1}\bar{\mathbf{L}}_1^\intercal\mathbf{Z}\bar{\mathbf{R}} = -\mathbf{S}\mathbf{Z}\bar{\mathbf{R}} \tag{169}$$

where $\mathbf{S} = \bar{\mathbf{R}}_1(\mathbf{B}_1 - \lambda_0\mathbf{I}_{N-1})^{-1}\bar{\mathbf{L}}_1^\intercal$, and it is called the group inverse of $(\bar{\mathbf{J}} - \lambda_0\mathbf{I}_N)$. It is intuitively a normalizing factor, and in this case $\mathbf{S} \approx -1/\lambda_0\mathbf{I}_N$ leads to Eq. (66) that we used in this work. The equation for the dominant left eigenvectors' first-order perturbation can be derived analogously. More rigorous and thorough mathematical derivations with regard to the eigenvectors can be found in [3], section 3.

References

1. Dunford N, Schwartz JT, Badè WG, Bartle RG. Linear Operators: Self Adjoint Operators in Hilbert Space. Interscience Publishers; 1963.

2. Schuessler F, Dubreuil A, Mastrogiuseppe F, Ostojic S, Barak O. Dynamics of random recurrent networks with correlated low-rank structure. Physical Review Research. 2020;2(1):013111.

3. Greenbaum A, Li Rc, Overton ML. First-order perturbation theory for eigenvalues and eigenvectors. SIAM review. 2020;62(2):463–482.
