# Supplementary figures and images for: Relating local connectivity and global dynamics in recurrent excitatory-inhibitory networks
Source: PLoS Comput Biol. 2023 Jan 23;19(1):e1010855. doi: 10.1371/journal.pcbi.1010855 (PMC9894562; doi:10.1371/journal.pcbi.1010855)

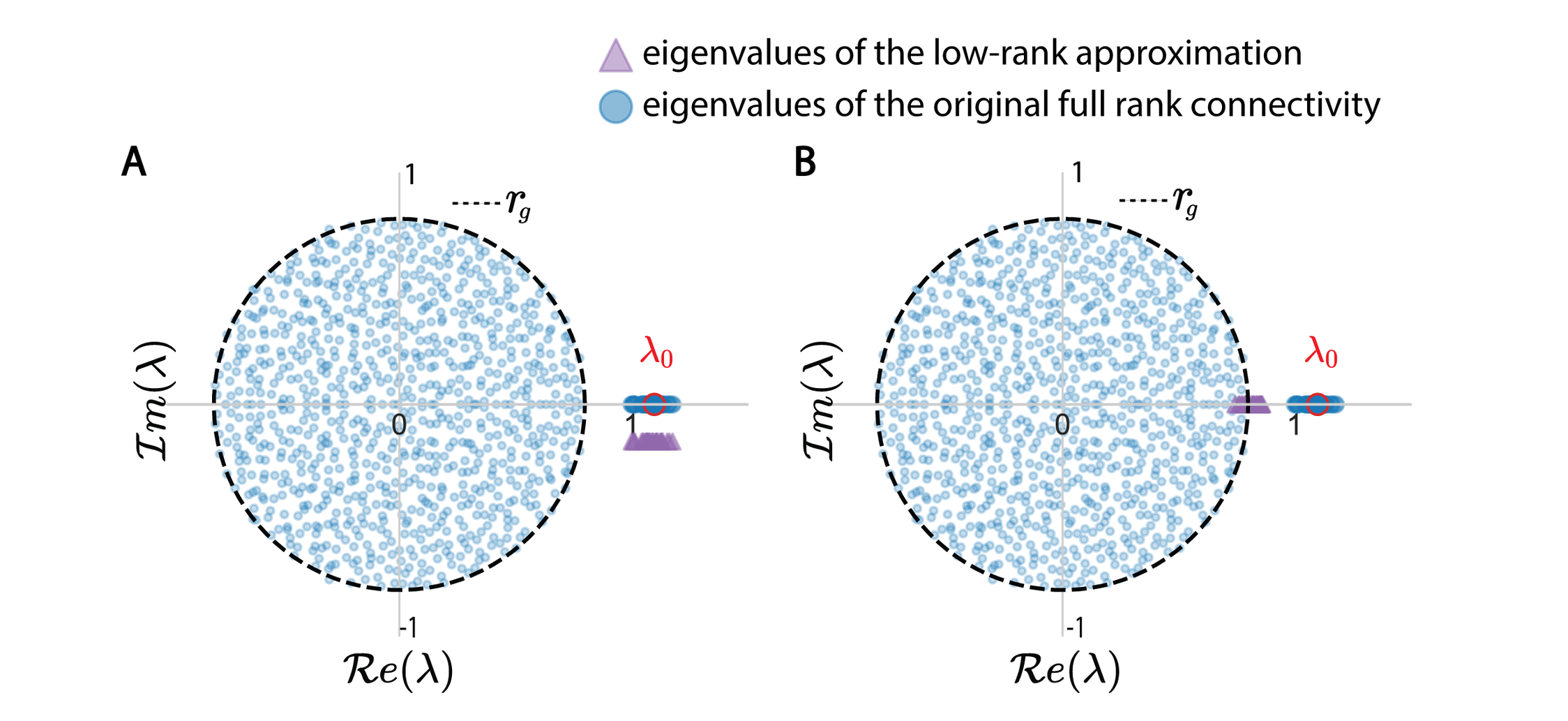

Supplement: S1 Fig — Blue scatters in (A, B) show eigenvalue spectra of the Gaussian excitatory-inhibitory full rank matrices J, with in general rank-2 mean connectivity J¯, and i. i. d. random parts with identical variances g2/N over neurons. Blue dots in the circular bulk show N − 1 complex eigenvalues for one realization of the random connectivity, outlying eigenvalues (blue dots) are shown for 30 realizations of the random connectivity. Dashed envelopes indicate the theoretical predictions for the radius rg = g of the circular bulk, red circles represent one of the eigenvalue of J¯ corresponding to the outlier of J. Network parameters NE = 2NI = 600, N = NE + NI, g = 0.8, J¯EE=0.0018,J¯IE=0.0015, J¯EI=0,J¯II=-0.0013. Purple triangles in (A) show the eigenvalues of the eigendecomposition-based rank-one approximation for the corresponding 30 realizations of the full rank matrices. Their location on the y-axis is shifted to help visualization. Purple triangles in (B) show the eigenvalues of the SVD-based rank-one approximation for the corresponding 30 realizations of the full rank matrices. (TIF) [file pcbi.1010855.s007.tif]
