# Supplementary material for: Relating local connectivity and global dynamics in recurrent excitatory-inhibitory networks
Source: PLoS Comput Biol. 2023 Jan 23;19(1):e1010855. doi: 10.1371/journal.pcbi.1010855 (PMC9894562; doi:10.1371/journal.pcbi.1010855)
Supplement: Attachment — Submitted filename: ReviewShaoOstojic.pdf [file pcbi.1010855.s008.pdf]

This is a great article for several reasons.

First, it starts filling an important gap between the ‘low-rank Gaussian mixture’ networks – which are defined using constrained ‘global’ variables to allow for full analytical tractability of their nonlinear dynamics – with networks whose connectivity is structured according to local probability rules. The latter networks are both very relevant models for neuroscience and difficult to analyze. Crucially, the authors show that the simplifying assumptions such as independence between neurons made in the ‘low-rank Gaussian mixture’ description do not imply constraints that are as stringent in the local description of the connectivity (where correlations and non-Gaussianity are allowed).

Second, the authors put a lot of efforts to cover many different relevant examples of locally structured connectivity matrices, including E-I separation, reciprocal connectivity motifs and sparsity.

Third, the authors obviously already put a lot of efforts to polish the text.

I only have a few thoughts concerning points of discussion of these great results, and minor suggestions on the presentation that the authors could optionally consider if they would find them useful.

General thoughts:

1. To conduct their analysis, the authors match a full-rank network (consisting of a rank-one E-I separated connectivity and a random ‘perturbation’) to a low-rank network. Here, to do so, the authors truncate the full rank network using eigendecomposition, preserving the ‘dominant’ (i.e., of larger norm) eigenvalues. The authors do note in the discussion that the minimal-Frobenius-norm match between a full-rank and a low-rank matrix would require taking the singular value decomposition of the full matrix. The authors rightly stress that, by construction, the match between dominant will be better when using eigendecomposition. However, I am not sure about the following statement (line 473, ‘as these eigenvalues determine the autonomous dynamics in the network’. Indeed, in general, for nonnormal matrices, the global (i.e., among all eigenmodes) correlations between eigenvectors will shape the dynamics - and this is even true for matrices with random iid elements, see e.g. Chalker and Mehlig, Eigenvector statistics in non-hermitian random matrix ensembles, PRL 1998. Accordingly, an exciting part of the author’s own results is the clarification of how reciprocal connectivity motifs introduce correlations between the entries of m and n (eq. 70, and lines 802-805) that shape the dynamics beyond the contributions of the eigenvalues (paragraph 2.6.2). In other words, it seems to me that if it was possible to analytically compute the SVD of a correlated EI separated matrix to approximate it with a rank-one matrix, this rank-one matrix might lead a better estimation of the dynamics of the full network than the truncated eigendecomposition (by better capturing the structure of eigenvector correlations across the full network) –even if the estimation of the leading eigenvalue might be less accurate. On the other hand, to the best of my knowledge, it is even harder to get analytics for the leading singular values and vectors of an arbitrary matrix, than for the leading eigenvalues and eigenvectors of this matrix – which makes the latter approach more

productive. Do the authors agree, and if so, could this be clarified? Would this suggest that the approximations set forth in the current work – which are in principle extensible to many different types of random connectivities - are more accurate for networks which are not too non-normal (which may be linked to the normalization of the weights and the need for the random part of the connectivity to be a 'perturbation')?

2. It may be good to mention the differences and similarities to Ocker, Josic, Shea-Brown and Buice, Linking structure and activity in nonlinear spiking networks, Plos Computional Biology 2017. This article uses a diagrammatic approach to find an approximation to the nonlinear dynamics of networks with (in principle, arbitrary) structured connectivity. I believe the approach set forth in this new submitted work leverages some restrictions on the nature of the connectivity matrix (rank-one deterministic part perturbed by a random matrix) to get more interpretable equations that do not require to determine which terms to keep in a diagrammatic expansion.

3. It might be worth discussing the fact that rate units could correspond to populations of (biological, spiking) neurons, rather than to individual neurons (along the lines of e.g. Ostojic and Brunel, Plos computational biology 2011; Gerstner, Kistler, Naud and Paninski, Neuronal Dynamics, Cambridge University Press, 2014). This means that (i) the connectivity statistics observed in the brain and (ii) the statistics of connections between rate units in a model, may be approximately linked in a very indirect -and possibly not bijective- way (e.g., in some cases, the E-I separation is lost in this mapping, as in Ch. 16.3, Dynamics of decision making, in Gerstner, Kistler, Naud and Paninski, Neuronal Dynamics, Cambridge University Press, 2014). In this context, the authors' major achievement of elucidating the dynamics of structured networks overcomes one of the challenges along the road of studying more biologically realistic neuronal networks.

4. A surprising and powerful result from this work is that the low-rank Gaussian mixture framework is less restrictive than one might have expected (it can handle correlations and non-Gaussianity in the locally-defined connectivity). The following is simply a suggestion to discuss a follow-up question that naturally comes up to me – but touching on this question is not needed, this is simply a potentially nice addition. Specifically, I am wondering whether the authors have some insights into the type of locally defined connectivity which would not be well-accounted for in the Gaussian mixture framework (besides, trivially, the fact that the possibility of a low-rank approximation is helpful to simplify the dynamics)? For instance, what might be lost when excluding correlations between neurons in the low-rank Gaussian mixture framework – the possibility to describe networks with some types of strongly correlated global structure?

<u>Suggestions on the presentation of the results:</u>

1. The authors clearly did a lot of work and present a large amount of results. The authors clearly made extra effort in awareness that this type of work comes with some challenges for presenting the results. What follows is really just a question of personal preference, so it may not be a useful suggestion and the authors should feel free to ignore it. There are several figures, and figure parts, which are repetitions of the same analyses done in different types of networks (e.g. Figs. 2-4). For me, it would help to add a 'reminder' of the type of network we're looking at, on the left of each 'line' of each figure (e.g. for Fig. 2, A-E, something along the lines of 'E-I with unstructured pert.'). Along these lines, while it is great to have data for a lot of network types, one possibility would be to select a subset of these different types for the main figures, and show the additional network types in supplementary figures.

2. For the people that are unfamiliar with the low-rank Gaussian mixture networks, I would like to make a suggestion that I feel may help people to go above the technicalities and to quickly grasp the results. In the main text and/or in the methods, I would insist that while there are major simplifying assumptions made in the low-rank Gaussian mixture networks -- namely that the connectivity components are drawn independently between neurons from Gaussian distributions – the surprising results of the authors is that these assumptions do not restrict the locally-defined connectivity to the same extent (it does not need to have independent or Gaussian entries). In other words, the low-rank Gaussian mixture framework is less restrictive than it may intuitively appear to be at first sight.

<u>Miscellaneous:</u>

- Line 340: you mention 'the variance of the steady-state of the population dynamics'; I think it refers to variance across neurons (rather than across time); could the authors confirm or correct and clarify in the text?

- line 154: I would already mention that the CLT works under some constraints on the synaptic weights – as the authors discuss very well in detail further later in the text

- Even though I was able to follow the procedure to create the sparse matrices (in sections 2.1.1 and 2.1.2), I was unable to grasp the procedure to form the Gaussian connectivities (i.e. when either simulating or analyzing the matrices using what I understand from the procedure in lines 579-586, I was unable to get the right statistics). This misunderstanding will be solved when the code will be accessible anyway, but it might be worth going over the paragraph again (e.g., is Y' Gaussian iid as stated on lines 580/581, or is it a 'linear combination' as indicated on lines 581/582?).

- Doesn’t Eq. 69 assume independence between the different z_ij? Isn’t this assumption violated in the case of the reciprocal connectivity motifs?

- Eqs 94-95: 'Similarly, for the covariance of each population we have' -> do you mean that you heuristically substitute lambda_0 for lambda in the equations of the covariance?

- In S5 appendix and in eq. 41, it may be nice to insist that this is the N -> infinity limit, and to stress that these results make use of the assumption of independence between neurons.

- Line 889: 'we further consider $\bar{n}$ p given by Eqs.' -> do you mean 'we further consider the Eqs. ‘?

- Eq 70: bar{m} is back, even though I believe it was omitted earlier –e.g. eq. 67 - because its elements are 1 in the normalization chosen by the authors (eq. 52)

- Eq 124: Should the zij always be put withing the expectation signs? These terms get dropped anyway, so it does not matter in the end.

- Similarly, in Eq. 126, I believe you may have an extra delta_ij

- Line 934, it is stated that ‘Here we replaced $\bar{m}$ p = 1’, but that does not seem consistent with latter equations (Eq. 128)

- Line 977: ‘we remove symbol [·]' -> why is that?

- It is not easy to match the (clear) notation of the author with the article about the perturbative approach (ref. 47; eq. 72). This is probably outside of the scope of the article, but - optionally- if the authors are able to provide a match of their own eqs. 62-64 with one equation of ref. 47 (their theorem 2 /eq 6 ?), it could help the technically-inclined reader.
