# Supplementary material for: Relating local connectivity and global dynamics in recurrent excitatory-inhibitory networks
Source: PLoS Comput Biol. 2023 Jan 23;19(1):e1010855. doi: 10.1371/journal.pcbi.1010855 (PMC9894562; doi:10.1371/journal.pcbi.1010855)
Supplement: Attachment — Submitted filename: PlosCB_response_YS_SO.docx [file pcbi.1010855.s009.docx]

**Responses to reviewers’ comments**

We sincerely thank the reviewers for their constructive and thoughtful feedback. We have significantly revised the manuscript accordingly. Below, we summarize the major revisions:

1. We updated the abstract to stress our goal of tying biological experiment driven modelling with artificial recurrent neural network driven modelling, and to specify our study on reciprocal motifs.
2. We reorganized Figures 2-4 concerning the results of approximating the local connectivity with low-rank structure, and we adjusted the nearby text to better fit the figures;
3. We elaborated the derivations of the series formula of the eigenvalues, and gave an asymptotic sum of the geometric series *without truncation* (new S6 Appendix). We showed the outlier predictions obtained without truncation, and compared them with the outlier predictions obtained through truncating approximation (colour triangles and circles in the eigenspectra) in Figures 3, 4, 9;
4. We elaborated the derivations of the first-order perturbation of the eigenvectors in the newly added S6 Appendix;
5. We have extended and enhanced the discussion of limitations, motivations, innovations and future expectations of this work in response to reviewers’ comments.

Below, we provide detailed responses (in blue) along with the original comments (in bold and italic font). The line, equation, and section numbers are referring to the new manuscript file (unless otherwise stated), with changes highlighted in orange in the pdf.

**Comments to the Authors:**

**Please note here if the review is uploaded as an attachment.**

**Reviewer #1:**

Classic models in theoretical neuroscience rely on unstructured connectivity: either Erdos-Renyi (or block-ER) or independent Gaussian weights. Two lines of result have called these models into question. First, physiological and anatomical studies have shown that patterns of connectivity between neurons (motifs) are over-represented compared to their chance levels in Erdos-Renyi networks. Second, the division of neurons into discrete classes suggests a low-rank contribution to the connectivity. In addition, trained neural network models often exhibit a learned low-rank connectivity. Here, Shao and Ostojic relate the dynamics of these two classes of networks.

This is a good paper, with work of the high quality I’ve come to expect in PLOS Computational Biology. Its organization and presentation, however, need some improvement. I also have some clarifying questions about several points.

**Major**

***First, regarding organization: The text surrounding Figures 3 and 4 does not at all match the organization of the figures. For example, starting in section 1.4, we have the first reference to figure 3: 3A. It is followed by references to Figures 4F-H, then 3M. This suggests that either the figures or the text should be significantly reorganized. Having two separate organizations for the figures and text is confusing to the reader. (Related to this point, I think Figures 3 and 4 could also easily be separated into multiple figures.) As it stands, the reader has to hunt around for the relevant figure panel, both skipping forwards/backwards to the relevant figure, and then searching within the figure. Once at a figure, it is natural to look a bit ahead/behind in the panels - but the divorce between figure and text organization means that the next panels I look at have nothing to do with the nearby text that sent me to the figure, leaving me unnecessarily confused and wondering why these panels are in the same figure. I leave it to the authors to decide which to revise (figures or text) but please choose a consistent presentation order for this section. The relation between Figures 5-9 and the text is much more clear and was a relief.***

Thank you for this suggestion, and we apologize for the confusing placement of Figures 3 and 4. The panels in Figures 3 and 4 have been re-arranged. The panels of Figure 3 now display the results from networks with homogeneous reciprocal correlations (\eta=\eta_{pq}, p, q=E, I); and panels of Figure 4 now display the results from networks with heterogeneous reciprocal correlations. We also revised the text to better align the panel order with the text descriptions of the panels.

***Second, regarding motifs: Throughout, the authors study networks with an over-representation of reciprocal connectivity motifs. What about other two-synapse motifs? In terms of the linearized dynamics, expanding (I-J)^{-1} in powers of J yields an expansion where the reciprocal motifs are at the same order as convergent, divergent, and feedforward chain motifs, and those other motifs also impact the statistics of fluctuations (Hu et al. 2013 J. Stat. Mech, 2014 PRE, 2018 PRE, Recanatesi PCB 2019, Dahmen et al. bioRxiv 2020). Can networks with an over-representation of these other two-synapse motifs also be mapped on to a low rank Gaussian mixture network?***

Thank you for this comment and for pointing out these highly relevant works.

Yes, the networks with an over-representation of different kinds of two-synapse motifs can be mapped onto a low-rank Gaussian mixture network, but these motifs have different effects on the low-rank connectivity statistics and the low-dimensional dynamics. We have been examining these effects in follow-up work, but prefer not to include other motifs in the present paper to avoid overloading it.

We have added a corresponding paragraph in the discussion (lines 631-639):

*“In addition to the reciprocal motifs that we have focused on in this work, it has been demonstrated that other second-order motifs, including convergent, divergent, and chain motifs, have important effects on the statistics of fluctuations* [*(Hu et al., 2013, 2014, 2018; Recanatesi et al., 2019)*](https://www.zotero.org/google-docs/?XIGcfb)*. Specifically, recent studies have revealed that the dimensionality of the balanced networks and the norm of the continuous eigenvalue bulk are tuned differently by the statistics of various types of motifs* [*(Nykamp et al., 2017; Dahmen et al., 2020; Hu & Sompolinsky, 2022)*](https://www.zotero.org/google-docs/?TNXwIv)*. Examining the effects of these additional types of motifs on eigenvalue outliers and corresponding low-dimensional dynamics would be an important future step to connect models to large-scale electrophysiological recordings of the cortical microcircuits* [*(Seeman et al., 2018; Dahmen et al., 2020; Luo, 2021; Campagnola et al., 2022)*](https://www.zotero.org/google-docs/?iXjIe8)*.”*

***Third: Regarding the sparse network. In Eq. 36 for example, how do higher-order statistics of the synaptic weights in the sparse network scale with N? If p is O(1/N) but the non-zero connections have O(1) strength, I think the higher cumulants are all of O(1/N) also. These are neglected in the Gaussian model, so we’d expect the Gaussian model to fail for stronger weights similarly to the classic Gaussian mean field theory for spiking networks (Ostojic 2014). Is this the case? How does this affect the relation to a low rank Gaussian mixture network?***

Thank you for this question.

When mapping sparse networks to Gaussian ones, the key quantities are moments of J_{ij}. In the sparse networks, the synaptic strengths (AE, AI in manuscript) and network’s sparsity (c in manuscript, Eq. 28) are set up in such a way that the variance of J_{ij} scales as g^2/N (this is identical to [(Brunel, 2000; Ostojic, 2014)](https://www.zotero.org/google-docs/?ueTqWc)). The central moments of order k>2 scale as 1/N^{k/2}, so that they can be neglected in the large N limit. The scaling of the second moment is therefore identical to the Gaussian model, which ensures that universality results hold for the radius of the eigenvalue bulk, as well as for the central limit theorem used for low-rank eigenvector statistics in first-order perturbation theory.

When using low-rank Gaussian mixture networks, deviations from the theory (theoretical predictions given by low-rank models) occur not because of higher moments, but because perturbation theory assumes a small value of the scaling factor g in the variance of J_{ij}. This happens in both Gaussian and sparse networks, and we now clarify this in:

Lines 219-222, Fig 2E

*“The reason for this deviation from the theory is that perturbation theory assumes a small value of the scaling factor g in the variance of J_{ij}; as a result, the deviation also reflects the systematic error resulting from the first-order approximation.”*

Lines 440-441

*“In this case, the variance of the synaptic strengths J_{ij} scales as 1/N, as we assumed for Gaussian connectivity.”*

Lines 450-454

*“Previous works have shown that for such sparse networks where the variances of synaptic weights scale as 1/N, the bulk of the eigenvalues are distributed within a circle in the complex plane with a radius r_g determined by Eq. 150 (Fig 8A)* [*(Ostojic, 2014; Herbert & Ostojic, 2022)*](https://www.zotero.org/google-docs/?SuqDcw)*, as expected from the universality theorem for random matrices* [*(Tao et al., 2010)*](https://www.zotero.org/google-docs/?xDRVvN)*. ”*

The reviewer is correct that the same parameter is responsible for the phenomenon described in [(Ostojic, 2014)](https://www.zotero.org/google-docs/?6q2Lb0) because of an additional instability when the radius of the eigenvalue bulk becomes larger than unity (see example in figure above).

***Fourth, regarding terminology. Throughout, the authors refer to the left and right eigenvectors of an arbitrary matrix J. For J to have an eigendecomposition it must be diagonalizable. Is this guaranteed for the networks studied? And if so, aren’t the left and right eigenvectors equal? Or, are these the left and right singular vectors of the connectivity? If so, I’d suggest using that terminology for accessibility to a broader readership.***

Thank you for this question. We studied connectivity matrices with randomly generated entries. Such matrices can be diagonalized almost surely (with probability 1) on complex numbers, as the set of non-diagonalizable matrices is of measure 0. The matrices we consider are however not symmetric and therefore non-normal [(Murphy & Miller, 2009)](https://www.zotero.org/google-docs/?nFIkB5), so that the left and right eigenvectors are in general not identical. Instead, they form a bi-orthogonal set.

More specifically, the equation for the right eigenvectors is JR=R\Lambda, the equation for the left eigenvectors is (J.T) L=L\Lambda, the bi-orthogonality is (L.T)R=\delta (now: lines 786-792, Eqs. 44-47) ([(Horn & Johnson, 2012)](https://www.zotero.org/google-docs/?gXRe3p), Chapter 1).

In general, for non-symmetric matrices the left and right eigenvectors are not identical to the singular vectors (V and U in J = USV.T).

We have added this explanation and the corresponding references [(Horn & Johnson, 2012; Murphy & Miller, 2009)](https://www.zotero.org/google-docs/?EqnbCZ) in lines 780-785:

“*The connectivity matrices J that we studied have randomly generated entries. Such matrices can be diagonalized almost surely (with probability 1) on complex numbers, as the set of non-diagonalizable matrices is of measure 0. The matrices we consider are however not symmetric and therefore non-normal* [*(Murphy & Miller, 2009)*](https://www.zotero.org/google-docs/?8D2cg9)*, so that the left and right eigenvectors are in general not identical. Instead, they form a bi-orthogonal set* [*(Horn & Johnson, 2012)*](https://www.zotero.org/google-docs/?6EeGI8)*. Specifically,...*”

***Fifth, regarding matrix perturbation theory. Perturbations usually refer to a small parameter. Are there requirements on the norm of Z? Truncating in Eq. 6 seems to imply a small norm of Z, although this isn’t stated explicitly. How does this relate to the norm of J, and to the mapping from an excitatory-inhibitory or sparse network? I’m not sure that I would expect the random part to be weaker than the structured part in these cases.***

The reviewer is correct. In our case, the small parameter is the *variance* of the random part Z of the connectivity. This parameter directly controls the spectral radius (r_g, *S4 Appendix*) of Z, i.e., the maximal real eigenvalue belonging to the continuous eigenvalue bulk. The spectral norm of the matrix Z gives the upper bound on the spectral radius r_g, therefore, the variance parameter also controls it. The key assumption of our analysis is that this maximal eigenvalue of Z is smaller than the outlying eigenvalue of J that we aim to approximate (Eq. 4, Methods Sec. 2.2 Eqs 40, 41). In terms of spectral norms, this presumably translates to the spectral norm of Z, which gives the upper bound on the spectral radius, being smaller than the norm of \lambda of J_{R1}=mn.T/N *(Dunford & Schwartz, 1963)*. This condition holds both for the Gaussian E-I networks and for sparse networks. We have now added it explicitly to the text:

Lines 160-164, eigenvalue outliers

*“We next assume that the maximal eigenvalue of Z is smaller than the outlying eigenvalues corresponding to the low-rank approximation mn.T/N we aim to determine (Eq. 4, Methods Sec. 2.2 Eqs 40, 41) . This condition holds as long as the variance amplitudes g_{pq}^2/N of the random part Z of the connectivity are not too large* [*(Martí et al., 2018; Dahmen et al., 2020)*](https://www.zotero.org/google-docs/?SH2rUd) *(S4 Appendix).”*

Lines 178-181, eigenvectors

*“An approximate expression for the right and left connectivity vectors m and n of J corresponding to the outliers \lambda can be determined using first order perturbation theory*[*(Greenbaum et al., 2019)*](https://www.zotero.org/google-docs/?YQSDGq)*, where we treat the variance amplitudes g_{pq}^2/N of the random part Z of the connectivity as small parameters.”*

Lines 440-441, sparse networks

*“In this case, the variance of the synaptic strengths J_{ij} scales as 1/N, as we assumed for Gaussian connectivity.”*

**Minor points:**

***1.Figure 2E: Where are the red curves for \sigma^2_{m^E}, \sigma^2_{n^E}?***

We thank you for pointing this out. In Figure 2E, the variance of J_{ij} is uniform across all connections and given by g^2/N. And according to Eq. 90, the variance of m_i (n_i) is the same when i belongs to the Exc and Inh populations, that is \sigma_{m^E}^2=\sigma_{m^I}^2, \sigma_{n^E}^2=\sigma_{n^I}^2. The two curves in Figure 2E therefore overlap, and we have set the red curve (Exc) to be thicker to help visualization.

***2.Line 183/184: [\theta_k] = 0, for all k greater than 0? theta_0 is independent of Z, I think.***

Thank you for pointing this out, we have corrected it (line 209).

***3.Eq. 17: What is \alpha_p? I can’t seem to find a definition for it in the surrounding text. Is \sigma^p_{nm} = \sigma_{n^p m^p}?***

\alpha_p is the fraction of neurons belonging to population p={E,I}, yes, \sigma^p_{nm} is the population covariance between elements m_i and n_i with i belonging to population p. We have placed these frequently used notations in Table 1 (line 640 before the ‘Materials and methods’ section). We have added these lines (lines 346-348):

*“..., \alpha_p is the fraction of neurons belonging to population p, \sigma_{nm}^p is the population covariance between elements m_i and n_i with i belonging to population p (Table. 1).”*

***4.Line 319: When only the first term is present in the res of Eq. 17, it reduces to the classic Amari-Grossberg rate model, which seems worth mentioning.***

Thank you for the comment. We have added the references (lines 374-375):

*“...this reduced model actually corresponds to the classic Amari-Grossberg rate model* [*(S.-I. Amari, 1975; S. Amari, 1977; Cohen & Grossberg, 1983; Cowan et al., 2016)*](https://www.zotero.org/google-docs/?352DHp)*”*

***5.Figure 5C: blue and purple is a low-contrast color choice that makes this harder to read.***

We have changed the colours to orange and purple. Thank you.

***6.Figures 5 and 6D: the dark colors here were also hard to quickly distinguish.***

We have changed the dark red and blue to orange and cyan. Thank you.

***7.Figures 5 and 6: How does the bistability in \kappa manifest in activity of the underlying network?***

Thank you for this question. The latent dynamic variable \kappa captures the population-averaged mean activation \mu_x^p, and we have shown this in Methods Sec 2.6.1 Eq. 111 (old Eq. 109), given \bar{m}^p=1. We added this equation in the Results Sec 1.5 line 336, Eq. 17.

The bistability in \kappa indicates the bistability of population dynamics, i.e., the underlying network's activity exhibits a high and a low state (bistability).

***8.Eq. 20: It looks like J should be regular typeface on the right-hand side.***

Thank you for pointing this out. We have fixed it (now Eq. 22).

***9.Eq. 29: If square brackets denote the average over the distribution of J, what does ()_J denote?***

This symbol was redundant. We have deleted it (now Eq. 31). Thank you.

**Reviewer #2:**

This is a great article for several reasons.

First, it starts filling an important gap between the ‘low-rank Gaussian mixture’ networks – which are defined using constrained ‘global’ variables to allow for full analytical tractability of their nonlinear dynamics – with networks whose connectivity is structured according to local probability rules. The latter networks are both very relevant models for neuroscience and difficult to analyze. Crucially, the authors show that the simplifying assumptions such as independence between neurons made in the ‘low-rank Gaussian mixture’ description do not imply constraints that are as stringent in the local description of the connectivity (where correlations and non-Gaussianity are allowed).

Second, the authors put a lot of efforts to cover many different relevant examples of locally structured connectivity matrices, including E-I separation, reciprocal connectivity motifs and sparsity.

Third, the authors obviously already put a lot of efforts to polish the text. I only have a few thoughts concerning points of discussion of these great results, and minor suggestions on the presentation that the authors could optionally consider if they would find them useful.

General thoughts:

***1. To conduct their analysis, the authors match a full-rank network (consisting of a rank-one E-I separated connectivity and a random ‘perturbation’) to a low-rank network. Here, to do so, the authors truncate the full rank network using eigendecomposition, preserving the ‘dominant’ (i.e., of larger norm) eigenvalues. The authors do note in the discussion that the minimal Frobenius-norm match between a full-rank and a low-rank matrix would require taking the singular value decomposition of the full matrix. The authors rightly stress that, by construction, the match between dominant will be better when using eigendecomposition. However, I am not sure about the following statement (line 473, ‘as these eigenvalues determine the autonomous dynamics in the network’. Indeed, in general, for nonnormal matrices, the global (i.e., among all eigenmodes) correlations between eigenvectors will shape the dynamics - and this is even true for matrices with random iid elements, see e.g. Chalker and Mehlig, Eigenvector statistics in non-hermitian random matrix ensembles, PRL 1998. Accordingly, an exciting part of the author’s own results is the clarification of how reciprocal connectivity motifs introduce correlations between the entries of m and n (eq. 70, and lines 802-805) that shape the dynamics beyond the contributions of the eigenvalues (paragraph 2.6.2). In other words, it seems to me that if it was possible to analytically compute the SVD of a correlated EI separated matrix to approximate it with a rank-one matrix, this rank-one matrix might lead a better estimation of the dynamics of the full network than the truncated eigendecomposition (by better capturing the structure of eigenvector correlations across the full network) –even if the estimation of the leading eigenvalue might be less accurate. On the other hand, to the best of my knowledge, it is even harder to get analytics for the leading singular values and vectors of an arbitrary matrix, than for the leading eigenvalues and eigenvectors of this matrix – which makes the latter approach more productive. Do the authors agree, and if so, could this be clarified? Would this suggest that the approximations set forth in the current work – which are in principle extensible to many different types of random connectivities - are more accurate for networks which are not too non-normal (which may be linked to the normalization of the weights and the need for the random part of the connectivity to be a ‘perturbation’)?***

We thank the reviewer for the kind and insightful comments. Indeed, eigen-decomposition and singular-decomposition are two often used but distinct methods for extracting dominant network structures. Each method has pros and cons depending on the addressed question. As the reviewer pointed out, in this work we focus on autonomous dynamics, namely the transition to bistability, and in this case truncated eigen-decomposition appears to be the most appropriate method as illustrated below (note that we approximate both the eigenvalue and the eigenvectors, and therefore take into account their contribution). However, we completely agree that if we had focused on input-driven transients [(Bondanelli & Ostojic, 2020)](https://www.zotero.org/google-docs/?JqTCyr), SVD truncation (or other methods, see Valente, Pillow and Ostojic NeurIPS 2022 [(Valente et al., 2022)](https://www.zotero.org/google-docs/?nLMM9K)) may have been more accurate.

To illustrate why truncated eigendecomposition works best, we have compared it to truncated SVD in the case of independent random connectivity.

As seen in the figures below, using the top singular vectors to approximate J actually results in an inaccurate eigenvalue outlier (right, purple compared to blue) as previously noted in [(Benaych-Georges & Nadakuditi, 2012)](https://www.zotero.org/google-docs/?nQ9pBY), whereas using truncated eigendecomposition naturally preserves the outliers (left, y-axis of purple scatters is shifted for visualization). The SVD low-rank approximation, specifically in the example provided, yields an outlier that is smaller than the critical value 1, which might lead to an inaccurate prediction for the bifurcation to bistability.

.

We added this line and reference in the discussion (line 540-542):

*“An SVD-based approximation preserves the top singular values, but in general not the top eigenvalues (S1 Fig), and this can lead to an inaccurate approximation of autonomous dynamics* [*(Benaych-Georges & Nadakuditi, 2012)*](https://www.zotero.org/google-docs/?O2MWjq)*.”*

***2. It may be good to mention the differences and similarities to Ocker, Josic, Shea-Brown and Buice, Linking structure and activity in nonlinear spiking networks, Plos Computational Biology 2017. This article uses a diagrammatic approach to find an approximation to the nonlinear dynamics of networks with (in principle, arbitrary) structured connectivity. I believe the approach set forth in this new submitted work leverages some restrictions on the nature of the connectivity matrix (rank-one deterministic part perturbed by a random matrix) to get more interpretable equations that do not require to determine which terms to keep in a diagrammatic expansion.***

Thank you very much for pointing us to this paper. This work examines the effect of connectivity structure on the spiking activity correlations, and, in particular, focuses on the effect of non-linearity on higher-order cumulants of the dynamics. The key innovation is the use of Feynman diagrams. As we are not specialists of that technique, we are not sure about the details of similarities and differences with our approach, but it possibly provides another avenue to address similar questions.

We now mention this work in Discussion (lines 588-590):

*“Other techniques such as Feynman diagrams* [*(Ocker et al., 2017)*](https://www.zotero.org/google-docs/?eZmtR7) *may provide a way to further study the effects of structure on dynamics and in particular correlations.”*

***3. It might be worth discussing the fact that rate units could correspond to populations of***

***(biological, spiking) neurons, rather than to individual neurons (along the lines of e.g. Ostojic and***

***Brunel, Plos computational biology 2011; Gerstner, Kistler, Naud and Paninski, Neuronal***

***Dynamics, Cambridge University Press, 2014). This means that (i) the connectivity statistics***

***observed in the brain and (ii) the statistics of connections between rate units in a model, may be***

***approximately linked in a very indirect -and possibly not bijective- way (e.g., in some cases, the***

***E-I separation is lost in this mapping, as in Ch. 16.3, Dynamics of decision making, in Gerstner,***

***Kistler, Naud and Paninski, Neuronal Dynamics, Cambridge University Press, 2014). In this***

***context, the authors’ major achievement of elucidating the dynamics of structured networks***

***overcomes one of the challenges along the road of studying more biologically realistic neuronal***

***Networks.***

The relation between rate and spiking networks is indeed an important question. As pointed out by the reviewer, a common approach is to interpret each rate unit as a population of spiking neurons. An alternative is however to map each spiking neuron onto a rate unit, and therefore compare rate and spiking networks with an identical connectivity. In follow-up work from the team [*(Cimesa et al., 2022)*](https://www.zotero.org/google-docs/?WlAcDV), we have shown that using this approach, theoretical results in rate networks directly predict low-dimensional dynamics in spiking networks with identical low-rank connectivity. That provides a possible justification for interpreting the connectivity in rate networks directly in terms of experimentally measured connectivity statistics.

We now mention this in the discussion (lines 607-617):

*“To further incorporate experimental data on synaptic connectivity into recurrent network models, an important question is how networks of rate units used here relate to more biologically realistic spiking networks where neurons interact through discrete action potentials* [*(Ostojic & Brunel, 2011; Gerstner et al., 2014)*](https://www.zotero.org/google-docs/?P9YCIF)*. One approach for investigating this relationship is to map each spiking neuron onto a rate unit, and therefore compare rate and spiking networks with an identical connectivity matrix. Using this approach, recent work has shown that theoretical results in rate networks directly predict low-dimensional dynamics in spiking networks with identical low-rank connectivity* [*(Cimesa et al., 2022)*](https://www.zotero.org/google-docs/?qS027D)*. This provides a possible justification for interpreting the connectivity in rate networks directly in terms of experimentally measured local connectivity statistics.“*

***4. A surprising and powerful result from this work is that the low-rank Gaussian mixture framework is less restrictive than one might have expected (it can handle correlations and non-Gaussianity in the locally-defined connectivity). The following is simply a suggestion to discuss a follow-up question that naturally comes up to me – but touching on this question is not needed, this is simply a potentially nice addition. Specifically, I am wondering whether the authors have some insights into the type of locally defined connectivity which would not be well-accounted for in the Gaussian mixture framework (besides, trivially, the fact that the possibility of a low-rank approximation is helpful to simplify the dynamics)? For instance, what might be lost when excluding correlations between neurons in the low-rank Gaussian mixture framework – the possibility to describe networks with some types of strongly correlated global structure?***

A major limitation of the Gaussian mixture framework is that it cannot account for heavy tailed distributions of synaptic weights, which appear prominently in experimental measurements (see e.g. [(Song et al., 2005)](https://www.zotero.org/google-docs/?14r8PT)). Indeed, the result of Gaussian-mixture distributions relies on the central limit theorem, which assumes a finite second moment for the distribution.

In our preliminary understanding, excluding the correlations between neurons (m_i/m_j and n_i/n_j, i$\neq$j), precludes the possibility to describe networks with neurons or neuron clusters with particularly strong connections and in-/out-degree.

We have added discussion (lines 583-588):

*“It however holds only as long as the entries on the resulting approximation low-rank vectors satisfy the assumption of independent entries across neurons, and the distribution of synaptic weights satisfies the assumptions of the central limit theorem. This specifically rules out, for instance, strong synaptic weights that may be analogous to connectivity hubs, as well as the heavy-tailed distributions often found in experimental studies* [*(Song et al., 2005; Buzsáki & Mizuseki, 2014)*](https://www.zotero.org/google-docs/?GRfeGU)*.”*

Suggestions on the presentation of the results

***1. The authors clearly did a lot of work and present a large amount of results. The authors clearly made extra effort in awareness that this type of work comes with some challenges for presenting the results. What follows is really just a question of personal preference, so it may not be a useful suggestion and the authors should feel free to ignore it. There are several figures, and figure parts, which are repetitions of the same analyses done in different types of networks (e.g. Figs. 2-4). For me, it would help to add a ‘reminder’ of the type of network we’re looking at, on the left of each ‘line’ of each figure (e.g. for Fig. 2, A-E, something along the lines of ‘E-I with unstructured pert.’). Along these lines, while it is great to have data for a lot of network types, one possibility would be to select a subset of these different types for the main figures, and show the additional network types in supplementary figures.***

Thank you for this suggestion. We have added cartoons to the figures to show different types of networks, and we have re-organized Figures 3 and 4.

***2. For the people that are unfamiliar with the low-rank Gaussian mixture networks, I would like to make a suggestion that I feel may help people to go above the technicalities and to quickly grasp the results. In the main text and/or in the methods, I would insist that while there are major simplifying assumptions made in the low-rank Gaussian mixture networks -- namely that the connectivity components are drawn independently between neurons from Gaussian distributions – the surprising results of the authors is that these assumptions do not restrict the locally-defined connectivity to the same extent (it does not need to have independent or Gaussian entries). In other words, the low-rank Gaussian mixture framework is less restrictive than it may intuitively appear to be at first sight.***

Thank you for this suggestion. We have added new lines in discussion paragraph (lines 520-531):

*“Gaussian-mixture low-rank networks may seem to rely on major simplifying assumptions to make the dynamics more tractable and interpretable, such as Gaussian-distributed entries of the connectivity vectors that are independent across neurons. Our analyses however show that this class of models is nevertheless less restrictive than it may appear at first. Specifically, by using our analytical approach, we show that even if the distribution from which the locally-defined J_{ij} is sampled is not Gaussian (e. g. Bernoulli), the corresponding entries m_i, n_j on the connectivity vectors nonetheless converge to a multi-variate Gaussian distribution in the large network limit, provided the conditions of the central limit theorem are met. Importantly, our results for the network connectivity with reciprocal motifs show that the independent assumption for m_i (n_i) does not rule out the possibility of reciprocally-correlated synaptic weights (J_{ij}, J_{ji}) in the locally-defined connectivity.”*

Miscellaneous:

***- 1. Line 340: you mention ‘the variance of the steady-state of the population dynamics’; I think it refers to variance across neurons (rather than across time); could the authors confirm or correct and clarify in the text?***

Yes, it is the variance across neurons, we have clarified this in the text (now line 384). Thank you.

***- 2. line 154: I would already mention that the CLT works under some constraints on the synaptic weights – as the authors discuss very well in detail further later in the text***

Thank you for this suggestion, we have mentioned the constraints of applying CLT in this place (lines 187-188):

*“If the elements {z_{ij}} in Z are random samples drawn from a distribution with overall mean and finite variance, the central limit theorem holds and therefore…”*

***- 3. Even though I was able to follow the procedure to create the sparse matrices (in sections 2.1.1 and 2.1.2), I was unable to grasp the procedure to form the Gaussian connectivities (i.e. when either simulating or analyzing the matrices using what I understand from the procedure in lines 579-586, I was unable to get the right statistics). This misunderstanding will be solved when the code will be accessible anyway, but it might be worth going over the paragraph again (e.g., is Y’ Gaussian iid as stated on lines 580/581, or is it a ‘linear combination’ as indicated on lines 581/582?).***

We apologize for the misleading descriptions. Lines 698-700 (old: lines 581/582) missed an “of” after “a linear combination”, matrix Y is a linear combination of Y’ and Y’.T. We have re-written this sentence:

*“Then, in order to generate a matrix Y with reciprocal correlations \eta_{pq}, we form a linear combination of Y’ and its transpose Y’.T”*

We modified Eq. (33) to show this linear combination.

***- 4. Doesn’t Eq. 69 assume independence between the different z_ij? Isn’t this assumption violated in the case of the reciprocal connectivity motifs?***

In Eq. 71(old: Eq. 69) \sigma_{m}^2 assumes that z_{ij} in the same row i are independent of each other, \sigma_{n}^2 assumes that z_{ji} in the same column i are independent of each other.

Eq. 69 (old: Eq. 67) indicates these constraints

\Delta m_i = (1/\lambda_0)\sum_j z_{ij}	(Same row i)

\Delta n_i = (1/\lambda_0)(\sum_{j in NE} \bar{n}^E z_{ji}+\sum_{j in NI}\bar{n}^I z_{ji}) (Same column i)

In the case of the reciprocal connectivity motifs, the correlated synaptic weights hold between z_{ij} and z_{ji}, and the pairs are typically not in the same row or column. So in the large network limit (N->\infty) the independence assumption in Eq. 71 (old: Eq. 69) does not violate the setting of reciprocal motifs.

***- 5. Eqs 94-95: 'Similarly, for the covariance of each population we have' -> do you mean that you heuristically substitute lambda_0 for lambda in the equations of the covariance?***

Yes, we have clarified this in lines 947

*“We substitute \lambda for \lambda_0 in Eqs. (95) for the covariance of each population”*

Thank you.

***- 6. In S5 appendix and in eq. 41, it may be nice to insist that this is the N -> infinity limit, and to stress that these results make use of the assumption of independence between neurons.***

Thank you for these suggestions. We have addressed these assumptions:

Lines 767-768, 771

*“..., and the assumption that m_i, n_i are drawn independently between neurons from a multi-variate distribution is satisfied,... in the large network limit.”*

S5 lines 1169-1171

*“When a rank-R connectivity matrix satisfies the assumption that for each population where i belongs to, m_i, n_i are drawn independently between neurons from a multi-variate Gaussian distribution, we show that in the limit of large network,...”*

***-7. Line 889: 'we further consider n̄p given by Eqs.' -> do you mean 'we further consider the Eqs. ‘?***

Yes, we have rephrased this sentence (line 1021):

*“We further consider Eqs. 38, 54-56 to obtain \bar{n}^p here, ”*

Thank you.

***- 8. Eq 70: bar{m} is back, even though I believe it was omitted earlier –e.g. eq. 67 - because its elements are 1 in the normalization chosen by the authors (eq. 52)***

We have corrected this (now Eq. 72). Thank you.

***- 9. Eq 124: Should the zij always be put withing the expectation signs? These terms get dropped anyway, so it does not matter in the end.***

***- 10. Similarly, in Eq. 126, I believe you may have an extra delta_ij***

Thanks for your careful review. We have corrected them and re-written the content Eq. 126-128 (now)

***- 11. Line 934, it is stated that ‘Here we replaced m̄p = 1’, but that does not seem consistent with latter equations (Eq. 128)***

We have deleted \bar{m}^p (=1) in Eq. (128) (now Eq. (130)). Thank you.

***- 12. Line 977: ‘we remove symbol [·]' -> why is that?***

Unlike the locally-defined connectivity \bar{J}+Z, where Z represents the noise, we assume that the global vectors m and n in the low-rank networks are determined without noise, so we remove the symbol [].

***- 13. It is not easy to match the (clear) notation of the author with the article about the perturbative approach (ref. 47; eq. 72). This is probably outside of the scope of the article, but - optionally- if the authors are able to provide a match of their own eqs. 62-64 with one equation of ref. 47 (their theorem 2 /eq 6 ?), it could help the technically-inclined reader.***

Thank you for your interest.

We have added a new S6 Appendix to show the derivation of Eqs. 64-66 (old: Eqs. 62-64). They correspond to Eqs 7, 8 and Chapter 3.1 in Greenbaum et al.

We can give a brief match of the variables used in our method with those used in Greenbaum et al.

Unperturbed (zero) state:

In Greenbaum et al.’s work:

mean matrix: A0,

mean right eigenvector: x0; mean left eigenvector: y0,

single non-zero eigenvalue of A0 related to (x0,y0): \lambda_0

In our work:

mean matrix: \bar{J}

mean right eigenvector: \bar{R}; mean left eigenvector:\bar{L} Eq. 52 (old: Eq. 50)

single non-zero eigenvalue: \lambda_0

scaled mean right eigenvector: \bar{m}, scaled mean left eigenvector:\bar{n}, the scalings are given in Eqs. 49, 50 (old: Eqs. 47,48)

After perturbation

In Greenbaum et al.’s work

matrix : A

derivative of the dominant right eigenvector: x’, of the left eigenvector: y’

the dominant right eigenvector: x; the dominant left eigenvector: y

the dominant eigenvalue : \lambda

In our work

matrix : J

derivative of the dominant right eigenvector: \Delta R, of the left eigenvector: \Delta L

the dominant right eigenvector: R; the dominant left eigenvector: L

the dominant eigenvalue: \lambda

Scaled right eigenvector: m, scaled left eigenvector: n, the scalings are given in Eqs. 49,50

Eq. 74 (old Eq. 72) in our work corresponds to Eqs. (33,34) in Greenbaum et al’s work,

scaled \tilde{y} — \hat{n}; scaled \tilde{x} — \hat{m}

**Reviewer #3:**

The main contributions of the manuscript are (1) deriving a rank-1 approximation to recurrent connectivity defined by local connection statistics and reciprocal motifs and (2) using the rank-1 approximation to predict the dynamics in E-I networks. These results are very interesting and original, both conceptually and on a technical level. The manuscript is mostly very well written. My main concerns and suggestions are on the interpretation and discussion of the literature, as detailed below.

**Major:**

***1. When describing single neuron results such as Fig 2D, the authors referred to them as “predictions of the theory.” But this is potentially misleading because to obtain the eigenvector entries of the prediction, one needs to know the matrix Z. This usually means knowing the connectivity matrix J, under which case one can compute the exact eigenvector without using the perturbation theory. Note that the situation is very different for the results on the statistical level, such as Fig 2B, E. These can be directly calculated from the *local statistics* of J, making them true predictions from the theory. Similar issues also occur in the dynamic results, such as Fig. 6D. I suggest carefully distinguishing these two types of results throughout the manuscript including the abstract, and perhaps rephrasing the single neuron results other than “predictions.”***

We thank you for bringing this to our notice, we had not realized the phrasing was misleading.

We have now modified the language as follows, for example:

Lines 215-216, approximating connectivity

*“We further find that perturbation theory accurately accounts for the individual entries of the eigenvectors…”*

Lines 411-413, dynamics

*“...the perturbative term in the rank-one approximation accurately accounts for the heterogeneous activity of individual neurons in specific realizations of the connectivity Z”*

throughout the manuscript.

When describing this type of result, we stressed "in a given/specific realization of the locally-defined/random connection" at each place.

We revised the abstract:

*“We specifically apply this method to excitatory-inhibitory networks with reciprocal motifs, and show that it yields reliable predictions for both the low-dimensional dynamics, and statistics of population activity. Importantly, it analytically accounts for the activity heterogeneity of individual neurons in specific realizations of local connectivity.”*

***2. The conditions and limitations of the method should be more clearly discussed. For example, the rank-1 approximation of connectivity will not work when lambda lies within the bulk spectrum (line 410 should be moved/repeated upfront). The case when lambda is within the bulk is related to balanced EI input on average and has important implications on the dynamics (e.g., ref 44) and thus is worth discussing. For predicting dynamics with rank-1 connectivity, I expect the approach would not apply when the leading eigenvalue is negative (e.g., Fig. 4F), even if the approximation of the connectivity is accurate. It should also be made clear in the abstract that the study specifically focused on the reciprocal motifs rather than general types of motifs.***

Thank you for these constructive suggestions and comments.

We have revised the text to stress the assumption that \lambda lies outside of the bulk (now lines 160-164, Eqs. 6, 7):

*“We next assume that the maximal eigenvalue of Z is smaller than the outlying eigenvalues corresponding to the low-rank approximation mn.T/N we aim to determine (Eq. 4, Methods Sec. 2.2 Eqs 40, 41) . This condition holds as long as the variance amplitudes g_{pq}^2/N of the random part Z of the connectivity are not too large* [*(Martí et al., 2018; Dahmen et al., 2020)*](https://www.zotero.org/google-docs/?sAhzDF) *(S4 Appendix). ”*

Second, we agree that the connectivity-dynamics relationship in the network receiving balanced E-I

inputs is a very interesting and important direction that is worth discussing. In the networks we considered, the low-rank skeleton of the locally-defined connectivity matrix is fully specified by the mean E-I connectivity, the dominance of this low-rank E-I structure over other random structures nonetheless requires the dominant eigenvalue is outside of the bulk. Furthermore, it is required that the variance of the local random connectivity be small for proper implementation of perturbation theory and CLT for eigenvector statistics. This small variance, g^2/N, also indicates that the networks we were working on had smaller bulk radius than the eigenvalue of the low-rank structure. But we also found some interesting results: the appearance of the conjugated complex outliers, some outliers that were randomly distributed outside of the spectrum of sparse E-I connectivity (Fig. 8A right panel), these might be related to transient dynamics, oscillations etc studied in [(Rajan & Abbott, 2006; Tao, 2013)](https://www.zotero.org/google-docs/?BiNoL5). We have added these lines in the Discussions (lines 548-560):

*“The eigenspectrum of such matrices in general consists of a continuously-distributed bulk that is attributed to the random component, and discrete eigenvalues that generated by the low-rank excitatory-and-inhibitory mean connectivity* [*(Rajan & Abbott, 2006; Tao, 2013)*](https://www.zotero.org/google-docs/?dbFIiB) *.*

*When the mean of excitatory and inhibitory weights approximately cancel each another, the corresponding eigenvalue is small and resides within the bulk.*

*A number of works have examined the bulk of the eigenvalue spectrum for random matrices*

[*(Sompolinsky et al., 1988; Rajan & Abbott, 2006;Tao et al., 2010; Ostojic, 2014; Aljadeff et al., 2015; Martí et al., 2018; Clark et al., 2022; Hu & Sompolinsky, 2022)*](https://www.zotero.org/google-docs/?esDS3F)*, and showed that the obtained eigenvalue statistics have important implications for network dynamics such as spontaneous fluctuations* [*(Amit & Brunel, 1997)*](https://www.zotero.org/google-docs/?TS5xs9)*, oscillations* [*(Bimbard et al., 2016; Beiran & Ostojic, 2019)*](https://www.zotero.org/google-docs/?rKAN0y) *and correlations in asynchronous irregular activity* [*(Renart et al., 2010)*](https://www.zotero.org/google-docs/?b7ASA7)*.*

*In contrast, in this work, we focus on the parameter regime where the discrete eigenvalues are outliers and well separated from the eigenvalue bulk.*

*The outlying eigenvalues, and in particular the corresponding eigenvectors have to our knowledge received less attention.”*

The approach still works when the leading eigenvalue is real and negative and outside of the eigenvalue bulk. We illustrate in the figure below the numerical simulations of the eigenvalue spectra and dynamics, as well as our theoretical predictions, in example networks with a leading negative eigenvalue (using positive transfer function \phi(x)=1+tanh(x-1.5)).

Panel (A) shows the eigenvalue spectra of two example networks. These two networks have the same i.i.d. random connectivity component (theoretical prediction r_g=0.717, dashed circles), JE = 0.6, but differing mean inhibitory connectivity parameter JI (left 1.5, right 2.4). Both random networks have one negative outlier (outlying blue points), which is on average identical to the eigenvalue of the mean connectivity matrix \lambda_0 = JE-JI (red circles). Panels (B, C) show the fixed points of the latent variable \kappa in the rank-one approximation. The lines in (B) show the dynamics \dot{\kappa} as function of \kappa. The intersections with y=0 correspond to the stable fixed points. Panel (C) shows the contribution of mean connectivity to the latent dynamics. Solid lines JI = 2.4, Dash lines JI = 1.5. The latent dynamic \kappa is correctly predicted by our method, which captures the average neural activation across the population of neurons (panel D). It is possible to describe this low population-averaged activity state using only the mean connectivity. Nevertheless, the heterogeneity of the collective dynamics (the variance of population neuron activities), cannot be predicted by a model based on mean connectivity. Additionally, under this parameter regime, our model can still accurately map the firing rate of individual neurons in specific instances of random connectivity matrices (panels E, F).

We have specified the reciprocal motifs in the abstract:

*“We specifically apply this method to excitatory-inhibitory networks with reciprocal motifs, and show that it yields reliable predictions for both the low-dimensional dynamics, and statistics of population activity. ”*

Thank you.

***3. Related to point 2, the claims about the generality of the approach, unless directly demonstrated, should be refrained from being made. For example, in the abstract, “can in general be approximated by…”, line 503 in Discussion “can be directly extended to networks with additional structure,” line 519 “can be extended to various forms of local connectivity motifs,” and line 520 “can be applied more broadly to study experimentally-obtained …” I suggest reducing or rephrasing them as directions for future work and potential impact of the current study.***

Thank you for this suggestion. Note that some of the statements are based on extensions of this work that were not included in the present manuscript. We have nevertheless revised the language at places indicated by the reviewer.

We have revised the abstract:

*“We demonstrate that multi-population networks defined from local connectivity statistics for which the central limit theorem holds can be approximated by low-rank connectivity with Gaussian-mixture statistics.”*

We revised the discussion and added these lines (lines 631-639):

*“In addition to the reciprocal motifs that we have focused on in this work, it has been demonstrated that other second-order motifs, including convergent, divergent, and chain motifs, have important effects on the statistics of fluctuations* [*(Hu et al., 2013, 2014, 2018; Recanatesi et al., 2019)*](https://www.zotero.org/google-docs/?HacFoX)*. Specifically, recent studies have revealed that the dimensionality of the balanced networks and the norm of the continuous eigenvalue bulk are tuned differently by the statistics of various types of motifs* [*(Nykamp et al., 2017; Dahmen et al., 2020; Hu & Sompolinsky, 2022)*](https://www.zotero.org/google-docs/?F3zfAw)*. Examining the effects of these additional types of motifs on eigenvalue outliers and corresponding low-dimensional dynamics would be an important future step to connect models with large-scale electrophysiological recordings of the cortical microcircuits* [*(Seeman et al., 2018; Dahmen et al., 2020; Luo, 2021; Campagnola et al., 2022)*](https://www.zotero.org/google-docs/?Qganyd)*. “*

***4. When describing the numerical results/figures, it should be noted which part of the numerics corresponds to finite-size effects vs. systematic errors due to the approximations. It will also be helpful to explain (more prominently) the asymptotic properties, i.e., the orders of the variables such as m, n, theta_k as N-> inf.***

Thank you for the suggestion. We have modified the figure captions/results to clarify whether the numerics correspond to the finite size effects or the systematic errors of approximation. For example:

Fig 2A

*“Outlying eigenvalues are shown for 30 realizations of the random connectivity, and for different g their location on the y-axis is shifted to help visualization, the dispersion reflects finite-size effects.”*

Fig 3

*“In (B, E, F, H, I, K, M), the departure of the centres of the numerical simulation results from the theoretical predictions reflect the systematic errors due to the first-order perturbation approximation of eigenvalues and eigenvectors.”*

For the first-order perturbation of the eigenvectors, we add these lines (lines 862-864):

*“In order to guarantee stability in the large network limit, we assume that the variance of the local random synaptic weights is satisfying \sigma_{z_{pq}}^2 = O(1/N). Consequently, we have \sigma_{m^p}^2=O(1) and \sigma_{n^p}^2=O(1) (Eqs. 70, 71)”*

For the first-order perturbation of the eigenvalue, we clarify in these lines (lines 898-899):

*“Given the stability guarantee \sigma_{z_{pq}}^2=O(1/N), the asymptotic variance of the first-order perturbation of the eigenvalue \sigma_{\lambda}^2 becomes zero in the limit of large network”*

We have added a new S6 Appendix to discuss the series formula containing \theta_k and approximation for calculating eigenvalue outliers.

***5. The second part of the paper shows that the dynamics of a recurrent neural network can be approximated by only considering the leading eigenvector and ignoring its bulk spectrum. I find this result by itself very interesting. But a discussion is missing on whether this question or closely related ones has been studied in the literature, particularly the previous work by the authors.***

Thank you for your interest. We are not aware of previous work that explicitly considered only the leading eigenvector and neglected the bulk. Previous work from the lab [(Mastrogiuseppe & Ostojic, 2018)](https://www.zotero.org/google-docs/?EKKbv2) examined dynamics in networks with a superposition of low-rank structures and Gaussian random connectivity using a version of mean-field theory for random connectivity based on [(Sompolinsky et al., 1988)](https://www.zotero.org/google-docs/?MLATgp). Extending this type of MFT to the situation where reciprocal connections are present is however challenging [(Martí et al., 2018)](https://www.zotero.org/google-docs/?49EFvh). Moreover, in contrast to the case where connectivity is exactly low-rank, when the additional full-rank random part is present the mean-field theory describes only the steady-state activity (and linearized dynamics around it), but not the full dynamics as in Eq. 100.

The relationship between the approach of [(Mastrogiuseppe & Ostojic, 2018)](https://www.zotero.org/google-docs/?wp2cAl) and the present work is discussed in Detail in Methods, Section 2.6.3.

***6. Regarding literature: The first part on the perturbation eigenvalues is closely related to this paper on random matrix, which should be cited and discussed, e.g., in line 485: Tao 2013, Outliers in the spectrum of iid matrices with bounded rank perturbations. The relation between second-order motifs and the low rank component of connectivity and outliers in the covariance spectrum has been studied in ref 62. This should be discussed, e.g., in line 484, 505. The biostability dynamics due to local statistics and motifs is conceptually related to Nykamp et al. PRE 2017 Mean-field equations for neuronal networks with arbitrary degree distributions, which should be cited.***

Thank you for these suggestions.

We now mention the classic reference [(Tao, 2013)](https://www.zotero.org/google-docs/?8oe9Q1) (lines 561-565)**:**

*“The techniques used in this work on the perturbation eigenvalues can be traced back to the classic work of Tao* [*(Tao, 2013)*](https://www.zotero.org/google-docs/?tV6IxT)*. The intriguing finding that low-rank perturbations on the i.i.d. matrices have a range of nonlinear effects on the outliers inspired our investigation:*

*If we think of the correlated random connectivity as perturbations on the low-rank dominant structure, what effects do these have on the outliers?”*

Thank you very much for the note on ref 62. We very much admire this work. For the low-rank component and outliers, it extended the conclusion in Tao 2013 to the covariance matrix of the network dynamics C (not the connectivity matrix J), where the original connectivity matrix J has homogeneous, i.i.d Gaussian entries; for the second-order motifs, they focused on their impact on the bulk eigenvalue spectrum of the covariance dynamics. To the best of our knowledge, they didn't connect these two aspects to investigate how motifs control outliers.

We have added a brief note in the discussion (lines 565-572)**:**

*“Using linear response theory and mean-field techniques, recent research* [*(Hu & Sompolinsky, 2022)*](https://www.zotero.org/google-docs/?aOUP25) *expanded Tao’s findings to network dynamics by investigating the outliers of the covariance matrix of the dynamic fluctuations in i.i.d. networks. In particular, that work showed the effects of second-order motifs on the eigenvalue bulk distribution of the covariance matrix.*

*Our analyses use the low-rank excitatory-and-inhibitory mean structure to go one step further and analytically demonstrate the impact of cell-type-dependent reciprocal motifs on the outlier \lambda (Methods Sec. 2.4.2).”*

We have added the reference (lines 598-599)**:**

*“The effect of changes to the underlying connectivity is to further regulate the internal network dynamics, i.e., the bistability transition* [*(Nykamp et al., 2017)*](https://www.zotero.org/google-docs/?8Te4Yi)*.”*

Thank you.

**Minor:**

***1. The Jbar in Eq. 3 is also a naive low rank approximation to J by discarding the noise part Z. Many results could benefit from a discussion using Jbar as a benchmark. This has been done in the dynamics part, such as line 304, 328. But the comparison may be worth adopting explicitly throughout the manuscript.***

Thank you for this suggestion. We have stressed and discussed the comparison between these two approximations throughout the results:

Lines 244-248

*“Although the mean connectivity establishes the network’s basic structure, the Gaussian-mixture low-rank approximation improves on it in terms of preserving connectivity fluctuations and reflecting the cell-type-dependent structural variances in the original locally-defined random connectivity part Z.”*

Lines 403-406

*“Specifically, the excitation-dominated dynamical regime is largely determined by the noiseless mean connectivity \bar{J}. but beyond this mean approximation, positive reciprocal correlations combined with the excitation-dominated connectivity enhance positive feedback with respect to mean connectivity alone (Methods Sec. 2.5.2, Eq. (97)).”*

***2. The difference in the leading eigenvalue perturbation for the iid Z case vs. reciprocal motif case should be emphasized (e.g., in the Discussion). For example, the truncation Eq.7 is, in fact, exact as N->inf for iid Z but is strictly an approximation with reciprocal motifs.***

***The rationale of truncating the expansion eq.6 at third order should be stated (it is the first nontrivial term for the reciprocal case).***

***Is it possible to have more than three outlier eigenvalues for the reciprocal motif case? It seems to be the case considering keeping higher order terms in Eq. 6.***

Thank you very much for bringing these to our attention. We realized that it would be helpful to go into more detail about how the series formula for the eigenvalue \lambda was derived, so we added a new S6 Appendix.

As the reviewer kindly pointed out, the reason we truncated up to the second order is because \theta_2 is the first nontrivial term for the reciprocal case. In the revised manuscript, we compare the results obtained using truncation approximation and using infinite series resummation in the example networks with reciprocal motifs (Figs 3, 4, and 9). We found that the truncation approximation predicts \lambda (outlier corresponding to \lambda_0) as accurately as the infinite series resummation. In addition, we also found that expression of the second order coefficient \theta2=\bar{n}.T Z^2 \bar{m}/N can be directly linked to the overlap of the low-rank vectors \sigma_{nm} = \bar{n}.T Z^2 \bar{m}/(N\lambda0^2) (Eq. 96), so we chose to truncate the series formula at third order. We also provided a detailed discussion in S6 Appendix. We also added these lines in the text (lines 166-173):

*“Although this nonlinear equation is a polynomial with infinite terms, there are at most finite N solutions for the eigenvalue outliers* [*(Schuessler et al., 2020)*](https://www.zotero.org/google-docs/?O7c7qY)*. More specifically, in this work, we are only focusing on the second-order reciprocal motifs in the random component. The second order coefficient \theta_2 in Eq. 7 is the first non-trivial term for the reciprocal case, so we truncate the series summation at k = 2 (included) to provide a more straightforward and understandable comprehension of how reciprocal motifs modify the eigenvalue outlier. In addition, we give the computation of eigenvalue outliers without truncation, and more elaborate explanations in S6 Appendix.”*

According to S6 Appendix Eq. 159 we agree with you that it is possible to have more than three outlier eigenvalues for the reciprocal motif case, but the specific set of parameters needs to be carefully determined.

***3. The complex eigenvalues of an asymmetric matrix such as J lack an intrinsic ordering. The author uses the absolute value to define the leading eigenvalue. But I am not sure this should always be the case. For example, when there are fewer reciprocal motifs, i.e., negative eta (similar to Fig. 9B), the elliptical bulk spectrum could exceed the magnitude of outlier lambda.***

Indeed in this work, we define the leading eigenvalue as the real eigenvalue with the largest absolute value. Under the Gaussian-mixture low-rank framework, we assume that the low-rank vectors are composed of real number elements. So here we are only concerned with the real eigenvalues and the intercept of the continuous eigenvalue bulk on the real axis (the largest real part of the eigenvalues in the bulk as r_g). We have paid less attention to the complex conjugated outliers. We termed them outliers because they can be distinguished from the eigenvalue bulk, and are visibly apart from other continuous complex eigenvalues.

***4. Please consider adding a reference to the equations when referring to terms or methods such as the “locally defined connectivity” to remind the reader (e.g., line 337).***

Thank you for this suggestion. We have paid attention to this and added the cross-references.

(e.g. now lines 384 (old line 337), 276-277, 285-287, 413, 455, 458, 464, 500, 510, etc.)

***5. It will be helpful to describe briefly the organization and logic of the sections (e.g., Gaussian connectivity first, then generalize to sparse connectivity) at the end of the introduction.***

Thank you for this suggestion. We have added a short paragraph to describe the organization of the paper (lines 52-63):

*“The paper is structured as follows. In Results Secs. 1.1 and 1.2 we explain our setup for the local and global representations of random connectivity, and introduce the low-rank approximation, matrix determinant lemma and perturbation theory which are the basis of our analytical approach. We first start from the case of Gaussian excitatory-and-inhibitory networks, Results Secs. 1.3, 1.4 demonstrate our analysis for approximating local connectivity with low-rank structure. In the following Results Sec. 1.5, we use the low-rank approximation models obtained to describe the nonlinear dynamics. Then, in Results Sec. 1.6 we generalize our analysis to sparse excitatory-and-inhibitory networks. Major novel findings, key insights, restrictions and future extensions are addressed in discussions. Technical details and a retour to the Gaussian-mixture low-rank framework are covered in methods, preceded by a table of notation (Table. 1)”*

***6. Eq. 4 should be referenced again between Eq. 5 and Eq. 6.***

We have added the reference and the sentence (lines 160-162):

*“We next assume that the maximal eigenvalue of Z is smaller than the outlying eigenvalues corresponding to the low-rank approximation mn.T/N we aim to determine (Eq. 4, Methods Sec. 2.2 Eqs 40, 41) . ”*

Thank you.

***7. In Fig. 3B, H, N, why do the theory curves for lambda_c1 and lambda_c2 not have exactly the same real part? They are complex conjugates from the third order polynomial, right?***

Yes, they share the same real part and are conjugate complex values. The real part of the eigenvalues are displayed in the upper panel, because the real parts of lambda_c1 and lambda_c2 are identical, the red and orange lines overlap.

***8. In Fig. 4 caption (E), k is not defined/referenced.***

We have clarified this in the figure caption. Thank you.

***9. It is worth mentioning in the summary of section 1.3 (its last paragraph) that for iid Z, lambda=lambda0.***

We have added this (line 249). Thank you.

***10. A brief derivation on how to apply ref 47 to get Eq. 9 would be helpful.***

We have added a new S6 Appendix to elaborate on these derivations. Thank you.

***11. Line 122, higher rank approximations?***

We have corrected it (now line 135). Thank you

***12. Line 360, “the major novel effect,” and other comparisons between iid and reciprocal motif cases should be discussed and summarized again in the Discussion for better visibility.***

Thank you for this suggestion. We have added sentences in Discussions:

Lines 569-572

*“Our analyses use the low-rank excitatory-and-inhibitory mean structure to go one step further and analytically demonstrate the impact of cell-type-dependent reciprocal motifs on the outlier \lambda…”*

Lines 578-581

*“In particular, these entries on the left and right vectors are uncorrelated in networks with i.i.d. local connectivity (Sec. 1.3); nonetheless, the reciprocal motifs further induce correlations between these entries on the left and right vectors and result in zero-mean overlaps \sigma_{nm}^p between the vectors for each population. (Sec. 1.4).”*

Lines 598-605

*“The effect of changes to the underlying connectivity is to further regulate the dimension of the internal network dynamics, i.e. the bistability transition* [*(Nykamp et al., 2017)*](https://www.zotero.org/google-docs/?CRPARG)*.*

*Particularly, in networks with i.i.d. random connectivity, even though the latent dynamics is primarily determined by the mean excitatory-and-inhibitory connectivity, the independent random part influences the heterogeneity of the neural activities and indirectly affects the bifurcation point (Sec. 1.5.1, Fig 5C); however, the reciprocal motifs in the random component directly control the latent dynamics by incorporating recurrent feedback with regard to mean connectivity alone (Sec. 1.5.2).”*

***13. Line 520, the ref 10 -> ref 22?***

We have corrected it (now line 634, ref 22; [(Trousdale et al., 2012)](https://www.zotero.org/google-docs/?EIdaHA) to [(Hu et al., 2013)](https://www.zotero.org/google-docs/?Hmo4TT)). Thank you.

Code can be accessed at

<https://github.com/shaonannan/Article-clean_relating_lr-local/tree/main/Figures_Separate>
